# Single-mode squeezed-light generation and tomography with an integrated optical parametric oscillator 

**DOI:** 10.1126/sciadv.adl1814

**Published:** 2024-03-13

**Authors:** Taewon Park, Hubert Stokowski, Vahid Ansari, Samuel Gyger, Kevin K. S. Multani, Oguz Tolga Celik, Alexander Y. Hwang, Devin J. Dean, Felix Mayor, Timothy P. McKenna, Martin M. Fejer, Amir Safavi-Naeini

**Affiliations:** ^1^ Department of Applied Physics and Ginzton Laboratory, Stanford University, Stanford, CA 94305, USA.; ^2^ Department of Electrical Engineering, Stanford University, Stanford, CA 94305, USA.; ^3^ Department of Physics, Stanford University, Stanford, CA 94305, USA.; ^4^ Physics and Informatics Laboratories, NTT Research Inc., Sunnyvale, CA 94085, USA.

## Abstract

Quantum optical technologies promise advances in sensing, computing, and communication. A key resource is squeezed light, where quantum noise is redistributed between optical quadratures. We introduce a monolithic, chip-scale platform that exploits the χ^(2)^ nonlinearity of a thin-film lithium niobate (TFLN) resonator device to efficiently generate squeezed states of light. Our system integrates all essential components—except for the laser and two detectors—on a single chip with an area of one square centimeter, reducing the size, operational complexity, and power consumption associated with conventional setups. Using the balanced homodyne measurement subsystem that we implemented on the same chip, we measure a squeezing of 0.55 decibels and an anti-squeezing of 1.55 decibels. We use 20 milliwatts of input power to generate the parametric oscillator pump field by using second harmonic generation on the same chip. Our work represents a step toward compact and efficient quantum optical systems posed to leverage the rapid advances in integrated nonlinear and quantum photonics.

## Teaser

A complex quantum optics experiment to generate and perform analysis on squeezed light is implemented on a single chip.

## INTRODUCTION

In the drive to improve the precision and performance of sensing systems, quantum optical phenomena are increasingly becoming indispensable (*1*, *2*). The essential resources in this respect are the squeezed states of light, which are quantum states that have reduced noise along one of their quadrature components (*3*). The development of integrated photonics, enabling the miniaturization of optical devices and systems on a chip scale, offers a natural avenue to further the capabilities of quantum optical systems and engineer them to address a broader range of real sensing problems (*4*). However, creating a portable and efficient squeezed-light source has remained a formidable challenge (*5*).

The generation of squeezed states of light can use resonant or nonresonant schemes, as well as either χ^(2)^ or χ^(3)^ optical nonlinearities (*6*, *7*). In bulk optics, resonant approaches with χ^(2)^ nonlinearities have been extensively demonstrated (*7*, *8*), have achieved remarkable levels of squeezing (*9*) and have been deployed in real quantum sensors (*10*). Resonant schemes for generating squeezing, i.e., the use of optical parametric oscillators (OPOs), offer several advantages: These methods squeeze the optical field with less pump power, they provide natural mode-matching, and they also enhance responsivity to signals in a way that can potentially combine with squeezing (*11*).

Advances in the field of nanophotonics have enabled the integration of OPOs on chip (*12*–*14*). The burgeoning interest and developments in quantum computing (*15*–*17*) have fueled the development of integrated OPOs for generating squeezed light. These demonstrations have used the χ^(3)^ nonlinearity to successfully generate squeezed light in nondegenerate mode, where the signal and idler are at different optical frequencies. For many quantum sensing and continuous variable quantum computing applications, degenerate operation of a squeezer is highly desirable. The use of the χ^(3)^ makes degenerate squeezing challenging as the nearly resonant and considerable circulating power of the pump field either requires the use of more complex cavity designs and pumping schemes (*18*, *19*) or operation at cryogenic temperatures for ponderomotive squeezers (*20*) to mitigate noise processes that arise.

Our work demonstrates degenerate squeezed-light generation and tomography by implementing an integrated photonic circuit incorporating an OPO. Our device works as a resonant χ^(2)^ squeezer, and we measure squeezing of 0.55 dB and anti-squeezing of 1.55 dB with 20 mW of input fundamental harmonic (FH) power. We demonstrate a monolithic chip that incorporates all essential components—bar the laser and two detectors—streamlining the system by reducing operational complexity, shrinking the size, reducing power consumption, and addressing environmental constraints.

## RESULTS

Our approach uses a periodically poled thin-film lithium niobate (TFLN) (*21*, *22*) and leverages its strong χ^(2)^ nonlinearity, electro-optic effect, and low-loss linear optical characteristics for the photonic circuit. Integrated TFLN devices have shown remarkable promise, offering a toolbox of capabilities such as efficient light generation spanning from mid-infrared to near-ultraviolet (*23*–*27*), dispersion engineering (*28*–*30*), electro-optic modulation and tuning (*31*, *32*), OPO (*13*, *14*), waveguide squeezers for efficient pulse squeezing (*33*) and with similar level of integration including on-chip pump generating section and homodyne measurement subsystem (*34*), and frequency combs (*35*, *36*). Here, we harness the full capabilities of this emerging platform, demonstrating a monolithic integrated quantum system for the generation and tomography of squeezed light.

**Fig. 1.**
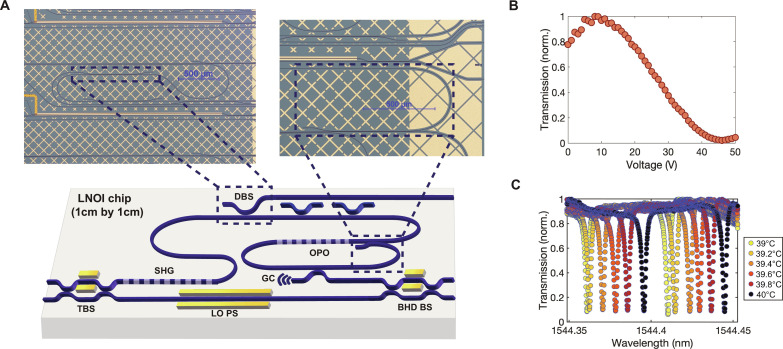
A monolithic quantum photonic integrated circuit for generating and analyzing squeezed light using a subthreshold OPO. (**A**) A monolithic quantum photonic integrated circuit (1 cm^2^ in area) on a periodically poled thin-film lithium niobate on insulator (LNOI) platform. The photonic integrated circuit consists of directional couplers, tunable beamsplitter (TBS), waveguide second harmonic generation (SHG), dichroic beam splitter (DBS), OPO, grating coupler (GC), local oscillator phase shifter (LO PS), and balanced homodyne beamsplitter (BHD BS). The top-left and top-right optical microscope images display the DBS for filtering out the FH after waveguide SHG, and a DBS in the cavity, respectively. Free space system is illustrated in fig. S9 for comparison. (**B**) The tuning behavior of the TBS with applied dc voltage where *V*_π_ ∼ 35 V (see the Supplementary Materials). (**C**) The tuning behavior of the mode spectrum of the OPO cavity at different thermoelectric cooler (TEC) temperature settings. The mode spectrum shifts by the entire free spectral range (∼50 pm) when we change the temperature setting of the TEC by 1.2°C.

Our device is a photonic integrated chip, with a surface area of roughly 1 cm^2^, that houses nearly all the required components to generate and analyze the squeezed vacuum state of light (Fig. 1A). We fabricated the chip on TFLN (see the Supplementary Materials). We couple FH light in the telecom C-band from a continuous-wave (CW) laser into the chip and then send it to a tunable beamsplitter (TBS). The TBS splits the light into two paths: One leads to the waveguide second harmonic generation (SHG) section to prepare the pump at the second harmonic frequency for the OPO, and the other serves as a local oscillator (LO). We adjust the division of light into these paths by tuning the dc voltage applied to the TBS electrodes. For light entering the SHG path, after propagating through the SHG section and generating frequency-doubled SH light, we filter the FH light out using three identical on-chip dichroic beam splitters. The SH light is then sent into another nonlinear section within a resonator and is used to pump an OPO to induce parametric gain in the cavity. This pump field only makes a single pass through the cavity due to the presence of the wavelength-dependent output coupler (*37*). As a result, below the oscillation threshold, a squeezed vacuum state at the FH emerges. For a balanced homodyne measurement, we combine the squeezed vacuum state and the prepared LO through the balanced homodyne beamsplitter (BHD BS). We adjust the dc voltage on the BHD BS electrode to balance the intensities of both outputs. By changing the dc voltage on the LO phase shifter, we can sweep the LO phase and measure the variance of the squeezed vacuum state across various reference phases.

A resonant cavity with parametric gain will oscillate once the gain exceeds the loss, forming an OPO. An OPO pumped such that its gain is lower than the oscillation threshold will parametrically amplify incident fields and thus generate squeezing. In principle, the achievable squeezing in such a system is only limited by the escape efficiency—the ratio between the rate at which photons that couple out of the device into the channel of interest divided by the total loss rate. Notably, while the amount of squeezing within the cavity is limited to at most 50%, the emitted output from the cavity can display arbitrarily large amount of squeezing due to an interference effect (*6*, *38*, *39*) in the limit of high escape efficiency.

**Fig. 2.**
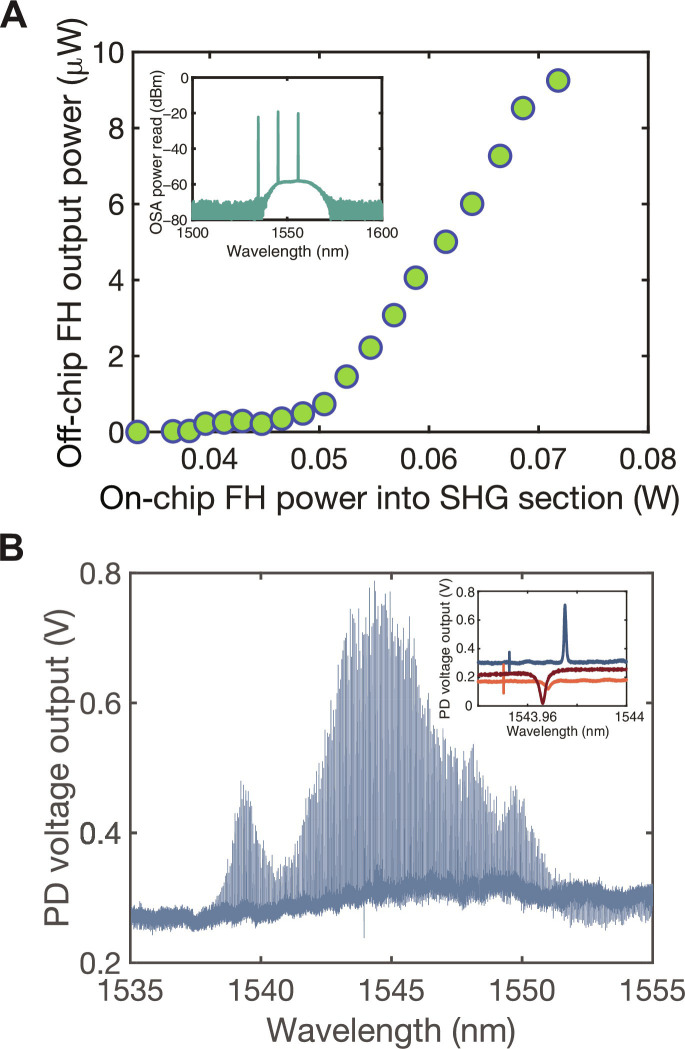
OPO characterization. (**A**) Output FH power at the output lensed fiber versus on-chip FH power going into the waveguide SHG section. We begin to observe oscillation at ~50 mW going into the SHG section where the inset shows the optical spectrum analyzer (OSA; Yokogawa AQ6370D) trace of the oscillation. (**B**) Cavity transmission in the presence of parametric gain (see the Supplementary Materials). We collect the voltage output of the photodetector (PD) versus the input laser's wavelength to the cavity with a parametric gain introduced by a second laser. Inset shows transmission at different pump power levels around the wavelength of the FH light used to generate gain. Red is the cold cavity transmission, orange is the cavity transmission at gain/loss = 0.62, and blue is cavity transmission at gain/loss = 0.88. We estimate the gain/loss ratio based on the linewidth of the modes where they were 2.84, 2.73, and 0.9 pm for red, orange, and blue trace, respectively (see the Supplementary Materials). Sharp line on the left of the resonance corresponds to the beating of the two lasers.

Two critical properties of the OPO are its oscillation threshold and its parametric gain spectrum. We characterize both. First, by performing a power sweep of the FH light entering the SHG section, we estimate the oscillation threshold of our OPO. We send FH power onto the chip, aligning its wavelength to the peak SHG response. This light is converted to SH, which then pumps the OPO. At sufficiently high power, the OPO oscillates and generates a substantial amount of FH light. We measure the total power in this OPO-generated FH light and its spectrum while sweeping the input FH power. We observe output FH power above the oscillation threshold corresponding to an input FH power exceeding ~50 mW. This corresponds to an on-chip SH power of 25 mW inferred from an independent waveguide SHG nonlinear conversion efficiency measurement (see the Supplementary Materials). A typical OPO spectrum is shown in the inset of Fig. 2A.

Second, we measure the gain spectrum of the OPO in the subthreshold regenerative amplifier regime (*40*, *41*) by observing the amplification of a second incident laser tone that we sweep across the cavity modes. Under typical circumstances, with no gain, cavity resonances appear as dips in the transmission spectrum due to the incident optical power being lost through the intrinsic loss channels. However, increasing gain begins to counteract this intrinsic loss, altering the transmission spectrum in the process. When the gain entirely compensates for the intrinsic loss within the cavity, the dips in the transmitted intensity vanish, signifying that the gain has compensated all loss. Any additional gain causes the amount of transmitted power to exceed the input and thus inverts the cavity line shape, leading to a transmission peaks with values surpassing unity. This is sometimes referred to as regenerative amplification (*40*). To make this measurement, we introduce a second laser through a separate grating coupler input path. This setup allows us to probe the transmission spectrum across different pump powers while sweeping the wavelength of the second laser. We observe peaks indicative of amplification as shown in Fig. 2B. The highest gain/loss ratio for modes in this plot is 0.88, as inferred from the mode’s linewidth (see the Supplementary Materials). We show the effect of increasing gain on a single mode line shape in the inset.

**Fig. 3.**
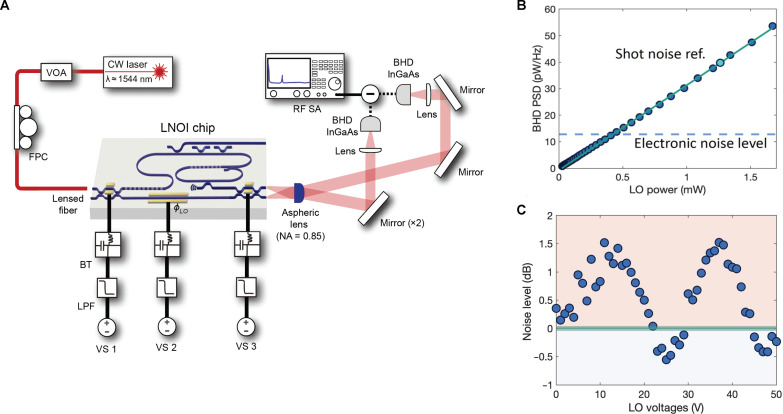
Squeezing measurement setup and results. (A) Squeezing measurement setup. CW light at 1544 nm from the low-noise laser is coupled into the chip using a lensed fiber. We control the input power going into the chip using a variable optical attenuator (VOA) and adjust the polarization using a fiber polarization controller (FPC). Three voltage sources (VC) are used to control the phase of the light where we use low pass filter (LPF) and bias tee (BT) to suppress non-DC signals going into the electrodes. We use high numerical aperture (NA) aspheric lens to efficiently collect light from the two output waveguides. The two beams are routed and focused onto the InGaAs photodetectors of the balanced homodyne detector respectively. We measure the power spectral density (PSD) of the difference between the two photocurrents using the RF spectrum analyzer (RFSA). (B) Measured PSD on the RFSA (electronic noise subtracted) averaged from 58 MHz to 60 MHz versus LO power. (C) Measured PSD on the RFSA (electronic noise subtracted) averaged from 58 MHz to 60 MHz versus dc voltage applied to the LO phase shifter at the TEC temperature of 43.4°C. On-chip FH power going into the SHG section was 20 mW.

After characterizing the performance of the individual components, we carry out the squeezing measurement using the setup shown in Fig. 3A. The output from the low-noise CW laser (ULN15PC, Thorlabs) goes through a variable optical attenuator and a fiber polarization controller before being coupled into the waveguide via a lensed fiber. We measure the coupling efficiency from the lensed fiber (SMF-28) to the input waveguide using a diagnostic waveguide (not shown in Fig. 1A), arriving at ∼32%, ignoring the propagation loss on-chip. Three voltage sources (PLH250-P, Aim-TTi) are used to apply dc voltages to the three electrodes on the chip, respectively. We observe a bias drift at a timescale slower than a millisecond, which may be corrected with feedback control schemes using thermo-optic phase shifters with high dynamic range in future sensing experiments. We actively lock to a bias point by feeding in the dc voltage readout from the BHD photodiodes to the voltage sources. We use external probes to connect the output of the voltage sources to the electrodes. We filter the voltages going to electrodes with low-pass filters (SLP-1.9+, Mini-Circuits) and bias tees (ZFBT-4R2GW+, Mini-Circuits). We collect the light emitted from the facets of the two output waveguides at the edge of the chip using a high numerical aperature (NA) aspheric lens (C037TME-D, Thorlabs). The two beams are collimated after the aspheric lens and propagate at slightly different angles. At a sufficient distance, the two beams are well separated. We use a dielectric mirror at this distance to route the two beams individually to each of the two InGaAs detectors of the BHD (HBPR-450 M-10 K-IN-FST, Femto). Last, we use plano-convex lenses (LA1134-C, Thorlabs) before the detectors to focus down the beam to the size of the photodiode. The total transmission from the output waveguide to the InGaAs detector was estimated to be 70%.

A precise shot noise reference is essential for calibrating the squeezing measurement. To obtain this reference, we collect the radio frequency (RF) spectrum of the balanced homodyne detector under balanced conditions while varying the incident optical power. We use the setup identical to the squeezing measurement scheme (Fig. 3A) for the shot noise measurement. To perform this measurement, the light is coupled into the input waveguide, and the TBS electrode is biased to send nearly all of the light into the LO path. The two optical powers incident on the two InGaAs detectors of the BHD detector are balanced using the BHD BS. A power sweep is carried out by varying the input FH power going into the chip. In Fig. 3B, the power sweep shows an ideal linear relation between the power spectral density (PSD) and the incident optical power. Here, we average the PSD measured on the RF spectrum analyzer (RFSA) over 2-MHz window centered at around 59 MHz. For all of the squeezing measurements, we use the real-time mode on the RFSA (FSW 8, Rohde & Schwarz) where we set the resolution bandwidth to 100 kHz and the video bandwidth to 28 MHz.

Having calibrated the shot noise level, we generate squeezed light with the OPO and use the LO phase to vary the quadrature that we measure to observe both squeezing and anti-squeezing. The LO power is fixed at 1.3 mW for this measurement. At this optical power, shot noise is 3.3 times larger than the total electronic noise level measured on the RFSA. The temperature setting of the thermoelectric cooler (TEC; Thorlabs TECF2S) is changed over 2.5°C centered around 42.5°C to go on and off the mode (see fig. S2). From an independent cavity transmission measurement, we verify that tuning the TEC temperature setting by 1.2°C tunes the mode spectrum over the free spectral range of the cavity (Fig. 1C). Separately, we sweep the LO phase electro-optically by applying a voltage to the LO phase shifter. We step the TEC temperature and collect RF spectra at each LO phase. We observe squeezing and anti-squeezing when the mode resonance wavelength aligns with the laser wavelength (see the Supplementary Materials). At 20 mW of on-chip FH power going into the SHG section, we measure squeezing of 0.55 dB and anti-squeezing of 1.55 dB (Fig. 3C).

**Fig. 4.**
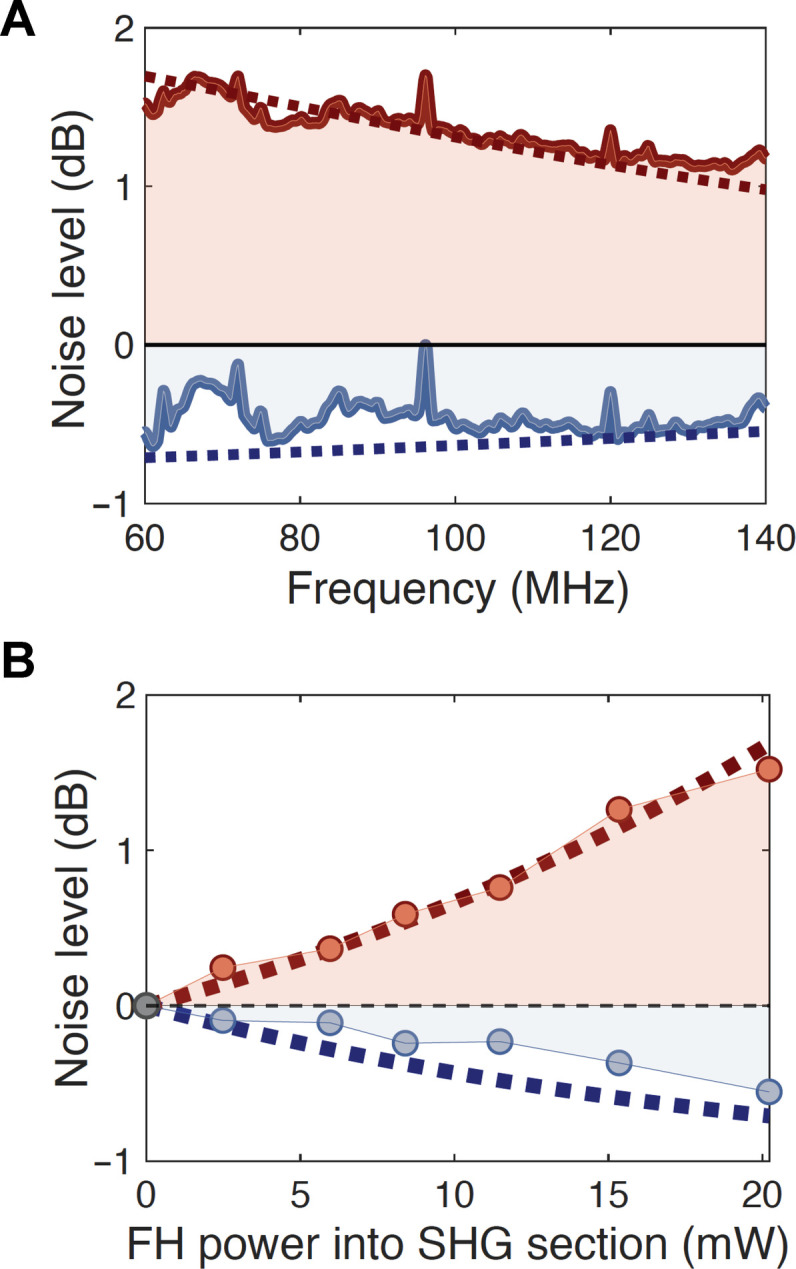
Squeezing spectra and power sweep. (**A**) Maximum (red) and minimum (blue) measured PSD spectrum (normalized by the shot noise, electronic noise subtracted) versus measurement frequency of the RFSA at the TEC temperature setting of 43.4°C with on-chip FH power of 20 mW going into the SHG section. Dotted lines represent the squeezing theory (see the Supplementary Materials). (**B**) Measured PSD values (normalized by shot noise, electronic noise subtracted) averaged from 58 to 60 MHz at around the peak (red) and dip (blue) of the LO phase shifter dc voltage sweep versus on-chip input FH power going into the SHG section. TEC temperature setting was at 43.4°C for this measurement. Dotted lines are theory lines (see the Supplementary Materials).

The bandwidth of squeezing and anti-squeezing from a cavity is limited by the linewidth of the mode of the cavity. In Fig. 4A, as we show that we are detuned from the cavity resonance, the amount of squeezing and anti-squeezing decreases. At 20 mW of on-chip FH power going into the SHG section, we measure squeezing and anti-squeezing spectra from 60 to 140 MHz. The frequency span of the measurement was limited by the RFSA. At frequencies below 60 MHz, we observe excess noise and are no longer limited by shot noise. These measured spectra align with the squeezing theory (see the Supplementary Materials).

In Fig. 4B, we perform a power sweep of the input FH and measure squeezing and anti-squeezing at different input powers. To perform this measurement, we first tune the TEC temperature to set the cavity frequency on resonance with the FH. At different input powers, we step the dc voltage applied to the LO phase shifter from 0 to 50 V and collect the RF spectrum at each voltage. We plot the measured PSD values around the peak and dip of the swing averaged from the measurement frequency of 58 to 60 MHz at different input powers. As we increase the input power, squeezing and anti-squeezing increase as expected (see the Supplementary Materials).

## DISCUSSION

Our work demonstrates the generation and tomography of degenerate squeezed states using an integrated OPO on TFLN. We have created a monolithic photonic circuit that integrates almost all essential components, enabling 0.55 dB of measured squeezing with only 20 mW of optical pump power. This represents an advance toward efficient and portable squeezed-light sources by harnessing the versatile χ^(2)^ nonlinearities available in emerging integrated photonics. When combined with lasers and detectors, our device can be developed into a complete system for deployable quantum-enhanced optical sensors. The three main sources of inefficiency in our system are as follows: a 35% escape efficiency, a 70% efficiency in the coupling from the output waveguide to free space, and a 75% quantum efficiency of photodiodes. Considerable improvements in squeezing levels are possible through further optimization of conversion efficiency, cavity *Q*, and escape efficiency and may enable the use of such devices in optical quantum computing (*42*–*44*). More specifically, enhancements such as a poled cavity with lower intrinsic loss, an over-coupled cavity with an extended racetrack-to-bus waveguide coupler, an aspheric lens with a low *f*-number composed of materials suitable for telecom applications (e.g., N-F2), a homodyne detector equipped with high quantum efficiency photodiodes, and an increase in on-chip SH power generation achieved either by developing an efficient fiber-to-waveguide coupler or by improving nonlinear conversion efficiency could lead to an extremely power-efficient integrated photonic squeezer (see the Supplementary Materials). With rapid progress in this burgeoning field, integrated resonant squeezers like the one created here will help unlock the full potential of quantum light sources across diverse applications.

## MATERIALS AND METHODS

### Design of the quantum photonic circuit

The waveguide geometry of the photonic integrated chip used in the OPO squeezing experiment follows our previous work (*14*), with a ridge waveguide width of 1.2 μm, height of 500 nm, and etch of 300 nm. The length of the poled section for both SHG and OPA is 1 cm, and there is an additional diagnostic SHG waveguide in the same poled section that is 6.5 mm long (not shown in Fig. 1A). Dichroic beamsplitters, which are 400 μm in length, allow the FH light to evanescently couple into the 1-μm-spaced adjacent waveguide while preventing the second harmonic light from traversing. The length of the racetrack resonator is ~2.2 cm, and it is designed to resonate the FH light and single-pass the second harmonic light (i.e., pump light). A dichroic beamsplitter closes the racetrack cavity for the FH and dumps the SH pump (Fig. 1A). The external coupler of the racetrack resonator is 120 μm in length and 1 μm in gap. The grating coupler is designed to operate at 1550 nm of maximum response at around the incident angle of 27°. The beamsplitters (TBS and BHD BS) consist of couplers that are 250 μm in length, and the lengths of the arms are around 2.7 mm. We place 2.5-mm-long electrodes next to one of the arms to induce phase shifts by applying dc voltages, and the gap between the waveguide and the electrode is 2.15 μm.

### Fabrication of the quantum photonic circuit

We start with an X-cut lithium niobate on insulator (NanoLN) chip with 500-nm-thick lithium niobate (LN) to fabricate the quantum photonic circuit. We first deposit 100 nm of SiO_2_ using a high density plasma enhanced chemical vapor deposition (Plasma Therm Versaline HDP CVD) tool. On top of the SiO2, we pattern Cr electrodes for periodic poling using electron beam lithography (JEOL 6300-FS, 100-kV)–based liftoff process. Applying high voltage pulses to these electrodes with a peak voltage of 700 V, we periodically pole the regions on the chip where SHG and OPA waveguides are placed. We were able to obtain consistent poling over the 1-cm-long section, verified by the SHG microscope (see fig. S3). After periodic poling, we remove the Cr electrodes using the Cr etchant and remove the 100-nm-thick oxide layer with buffered oxide etchant. We then pattern hydrogen silsesquioxane mask using electron beam lithography and etch the LN using an ion mill to make waveguides. We perform another electron beam lithography–based liftoff process to pattern 100-nm gold electrodes next to the waveguides where we aim to control the phase of the transmitted light. Using the HDP CVD system, we deposit 700 nm of SiO_2_ for cladding. Then, we deposit photoresist (SPR3612) and make patterns using photolithography for vertical interconnect access. We etch the SiO_2_ cladding down to the 100-nm gold electrodes to make vias using inductively coupled plasma etch system (Plasma Therm Versaline LL ICP Dielectric Etcher). After making vias, we pattern 200-nm-thick gold pads on the top surface of the chip to make contact with the probes. For this, we use the identical process that we used for making the 100-nm gold electrodes. Last, we anneal the chip in an oxygen environment at 500°C for 8 hours.

## Supplementary Materials

### This PDF file includes:

Supplementary Text Figs. S1 to S9 References
