# Supplementary material for: Single-mode squeezed-light generation and tomography with an integrated optical parametric oscillator
Source: Sci Adv. 2024 Mar 13;10(11):eadl1814. doi: 10.1126/sciadv.adl1814 (PMC10936947; doi:10.1126/sciadv.adl1814)
Supplement: Supplementary file 1 — Supplementary Text Figs. S1 to S9 References [file sciadv.adl1814_sm.pdf]

Science Advances
AAAS

Supplementary Materials for

Single-mode squeezed-light generation and tomography with an integrated optical parametric oscillator

Taewon Park *et al.*

Corresponding author: Amir Safavi-Naeini, safavi@stanford.edu

Sci. Adv. **10**, eadl1814 (2024)
DOI: 10.1126/sciadv.adl1814

This PDF file includes:

Supplementary Text
Figs. S1 to S9
References

1 Quantum photonic circuit and experiment details

1.1 Waveguide second harmonic generator characterization

To characterize the second harmonic generation section of our circuit, we start by measuring its spectral response. To access the waveguide SHG section, we couple light into the output port of the first DBS and collect the output at the top input waveguide (Fig 1.(a)). Since the coupler of the TBS does not couple SH light to an adjacent waveguide, we may take a wavelength scan of the fundamental harmonic to look at the spectral response at the second harmonic. In Fig S3.(a), we show the spectral response of the waveguide SHG section measured with this

method. To extract the nonlinear conversion efficiency, we use the additional diagnostic SHG waveguide (not shown in Fig 1.(a)). We perform calibrated power sweep at the wavelength that gives the peak SHG response. We plot the SH power on-chip versus FH power on chip in Fig S3.(b) where the quadratic fit gives normalized conversion efficiency of 1000%/W. Assuming negligible measurable propagation loss of the diagnostic waveguide, the SMF-28 lensed fiber to waveguide coupling efficiency was 32% at 1550 nm. Waveguide to SMF-28 lensed fiber coupling efficiency was 30% at 780 nm. Although the peak SHG response is near 1543 nm, we must consider the nonlinear conversion efficiency at the wavelength of our low-noise laser used for the squeezing measurement. Fig S3.(c) shows the normalized conversion efficiency near the wavelength of the low-noise laser (1544.4 nm). Assuming that the nonlinear conversion efficiency at the peak is identical for both the diagnostic SHG waveguide and the actual SHG waveguide, we can infer the normalized conversion efficiency based on the relative spectral response at different wavelengths. The normalized conversion efficiency was around 600%/W at 1544.4 nm using this analysis. We also observe that the SHG spectral response does not shift substantially as we change the TEC temperature setting over 2 °C, which is the temperature tuning range of our squeezing measurement.

1.2 Cavity characterization

To measure the cavity transmission, we couple light from the laser at the fundamental harmonic into the grating coupler using an angle-cleaved fiber (Fig S 5.(a)). We scan the wavelength of the tunable laser (Santec, TSL710) from 1500 nm to 1620 nm and collect the transmission. The transmission of the cavity is shown in Fig S5.(a), where the broad spectral response is from the grating coupler. Assuming that the cavity is undercoupled, a lorentzian fit to the mode near the low-noise laser wavelength gives Q_{int} of 950k and Q_{tot} of 550k. To determine the coupling condition of the cavity mode at the operating wavelength, we measure the phase response across

the optical resonance (*45, 46*). We park the laser near the optical resonance frequency and generate optical sidebands using an electro-optic modulator. Then, we measure the phase response of the optical to optical scattering parameter with the vector network analyzer (VNA). Shown in Fig S4 (b), the measured phase response shows relatively small change in phase across the resonance frequency, indicating that the mode is undercoupled. By fitting the measured phase response data, we verify that the mode is undercoupled.

1.3 Optical parametric oscillator characterization

We characterize the optical parametric oscillator using the measurement setup shown in Fig S5.(a). First, we estimate the threshold of our OPO using tunable laser 1. We vary the FH power that couples into the input waveguide and take a broad wavelength scan in the range of the EDFA gain (1530 nm to 1560 nm) at different input on-chip power levels. For each scan, we collect the voltage read on the InGaAs avalanche photodiode (APD, Thorlabs APD410C). We observe an abrupt increase in the voltage read on the APD when the OPO starts to oscillate. At the wavelength exhibiting the minimum oscillation threshold, we perform a high-resolution wavelength sweep to record the optical spectrum (inset of Fig. 2(a)). Next, we measure the cavity transmission in the presence of parametric gain using two CW lasers at the fundamental harmonic. To generate parametric gain in the racetrack resonator, we park the wavelength of tunable laser 1 at the peak waveguide SHG response. We tune the magnitude of parametric gain by changing the input fundamental harmonic power going into the chip with the VOA. At different levels of parametric gain, we take a wavelength scan using tunable laser 2 and collect the voltage read on the InGaAs APD. For the cavity transmission measurement, we use an angle-cleaved fiber to couple telecom light from the tunable laser 2 into the grating coupler. Fig S5.(b) shows the theory plot of the cavity transmission in the presence of parametric gain. As we increase the gain, the linewidth of the mode becomes narrower and at a high gain region

below threshold we observe peak due to amplification.

1.4 Electro-optic phase shifter characterization

To characterize the electro-optic phase shifter, we take broad wavelength scans at different DC voltages applied to the TBS electrode. We couple light from a tunable laser at the fundamental harmonic into the bottom input waveguide using a lensed fiber. Then, we step the voltage from 0 to 50 V and collect the output light using a lensed fiber from the BHD BS top output waveguide and the BHD BS bottom output waveguide in separate measurements. Assuming identical coupling efficiencies for both output waveguides, we add the two output powers to obtain the magnitude at the LO path after the TBS. In Fig 1.(b), we plot this sum at different voltages at a wavelength of 1520 nm and normalize by the peak transmission value to obtain V_π.

1.5 Fundamental harmonic transmission characterization of the dichroic beamsplitter

The dichroic beamsplitter (DBS) consists of a directional coupler with identical waveguides adjacent to each other. The length of this coupler is 400 μm and it is designed to transfer light at the telecom wavelength with 100% efficiency. The gap is set to 1 μm to prevent second harmonic light from evanescently coupling to the adjacent waveguide at the coupler section. The coupling coefficient of the directional coupler is wavelength-dependent, and the performance of the DBS deviates from 100% transmission at the low-noise laser wavelength (1544.4 nm) due to the fabrication imperfection. To characterize the transmission of the DBS at the fundamental harmonic, we couple light using a SMF-28 lensed fiber into the output waveguide from the first DBS (see Fig 1 .(a)). Then, we use a tunable laser (TSL-710, Santec) and take a broad wavelength scan. We collect light coming out from the two input waveguides, respectively, using an SMF-28 lensed fiber. To get the power at the waveguide between the TBS and the

DBS, we add the optical power at the two input waveguides. For this, we assume that the coupling efficiencies from both input waveguides to a lensed fiber are identical. In Fig S6.(a), the wavelength dependence of the inferred optical power at the waveguide after the light passes through the dichroic beamsplitter is shown. Here, the wavelength dependence of the coupling efficiency (measured from an independent measurement using the diagnostic SHG waveguide) is taken into account when we infer the power at the waveguide. We estimate the transmission of the DBS to be 94$\pm$2% at the low-noise laser wavelength, assuming 100% transmission at around 1580 nm. In Fig S6.(b), we show that the wavelength dependence of the coupling efficiency is minimal and that the wavelength dependence in Fig S6.(a) is due to DBS.

1.6 Laser noise characterization

We study the noise properties of the low-noise laser prior to our squeezing measurement. First, we characterize the intensity noise of the laser. To check that our laser does not have excess intensity noise, we take a power sweep and measure the photocurrent PSD from the photodetector at different incident optical powers using RFSA. If there is no excess intensity noise, the measured power spectral density (PSD) should have a linear dependence with respect to the incident optical power. Fig S7.(d) shows this linear relation, verifying that the laser has no measurable excess intensity noise. The measured photocurrent PSD spectrum at different incident optical power on the photodetector is shown in Fig S7.(c). Next, we characterize the phase noise of the laser. To measure the phase noise of the laser, we send the output of the laser to one of the input port of a 2 by 2 fiber Mach-Zehnder interferometer (MZI) with a known FSR of 67 MHz. One of the two output ports is routed to the photodetector and we measure the photocurrent PSD spectrum using the RFSA. Shown in Fig S7.(e), the measured phase noise aligns with the theory that assumes a laser linewidth of 100 Hz, which is the reported value from the manufacturer.

1.7 Loss in our measurement and the anticipated on-chip squeezing with improved components

The measured squeezing in our experiment is limited by the loss in our OPO resonator, the loss after the output waveguide, and the non-ideal nonlinear conversion efficiencies on the chip. Our racetrack resonator has intrinsic quality factor of 950k where intrinsic quality factors of 10 million have been demonstrated on the same platform (*47*). There are two major sources of loss after the output waveguide: insertion loss of the aspheric lens and low quantum efficiencies of the photodiodes of the balanced homodyne detector. The insertion loss of our aspheric lens is over 20% at the telecom wavelength due to the material absorption (BD-2 glass). Using an aspheric lens with a material that has lower loss at the telecom wavelength (e.g., N-F2) and smaller f-number, we expect the coupling efficiency to be over 90%. The quantum efficiencies of the InGaAs photodiodes of our balanced homodyne detector were around 75% at the telecom wavelength, where balanced homodyne detectors with InGaAs photodiodes of quantum efficiencies over 99% have been utilized in squeezing experiments (*9*). Normalized conversion efficiency at the peak SH response was around 1000%/W for a 1-cm-long PPLN waveguide on our chip. The spectral response illustrated in Fig S3 (a) exhibits a wider distribution and diminished peak efficiency compared to theory. By analyzing the integral of this response, we infer that minimizing inhomogeneity could potentially improve the peak efficiency to 3000%/W-cm^2, where efficiency of 4000%/W-cm^2 is theoretically feasible (*23*). Fig S8 shows anticipated squeezing and anti-squeezing with different normalized nonlinear conversion efficiencies, intrinsic cavity quality factors, and aggregate losses. In scenarios where we have a FH power of tens of milliwatts and a racetrack resonator with an intrinsic quality factor of 10 million, a non-ideal nonlinear conversion efficiency would not be as critical. Nonlinear conversion efficiencies greater than 2600%/W-cm^2 could achieve more than 10 dB of squeezing at the cavity's output with sub-20 mW input power. Squeezing is generally sensitive to the escape efficiency and

aggregate loss following the cavity's output. For a cavity with a total quality factor of 200k, an intrinsic quality factor of 1 million would result in approximately 6 dB of squeezing at the cavity's output at the input power of 16 mW limited by the escape efficiency. An intrinsic quality factor of 10 million would increase the level of squeezing to 16 dB.

2 Squeezing theory

2.1 System hamiltonian and equations of motion

The hamiltonian for a periodically poled doubly resonant cavity that consists of $2N$+1 modes around the fundamental frequency (ω_p) with a non-resonant pump at the second harmonic could be written as,

$$\begin{aligned}\frac{\hat{H}}{\hbar} = \quad & 2\omega_p \hat{b}^\dagger \hat{b} + \sum_{n=-N}^{n=N} \omega_n \hat{a}_n^\dagger \hat{a}_n + \sum_{n=0}^{n=N} (g_n \beta_n e^{-2i\omega_p t} \hat{a}_n^\dagger \hat{a}_{-n}^\dagger \\ & + g_n \beta_n e^{2i\omega_p t} \hat{a}_n \hat{a}_{-n})\end{aligned} \tag{1}$$

where ω_0 is the cavity resonance frequency closest to the fundamental frequency (ω_p). g is the nonlinear interaction rate and β is the pump field (for $n = 0$, $\beta_n \to 2\beta_0$). The Heisenberg equation of motion including the loss terms is then,

$$\begin{aligned}\frac{d\hat{a}_n}{dt} = \quad & -i\omega_n \hat{a}_n - i g_n \beta_n e^{-2i\omega_p t} \hat{a}_{-n}^\dagger - \frac{\kappa_n}{2} \hat{a}_n \\ & + \sqrt{\kappa_{n,e}} \hat{a}_{in}(t) + \sqrt{\kappa_{n,i}} \hat{a}_{in,i}(t).\end{aligned} \tag{2}$$

We now go into the frame of the fundamental harmonic where $\hat{a}_n \to \hat{a}_n e^{-i\omega_p t}$ and $\hat{a}_n^\dagger \to \hat{a}_n^\dagger e^{i\omega_p t}$. Equations of motion then becomes

$$\begin{aligned}\frac{d\hat{a}_n}{dt} = \quad & -i(\omega_n - \omega_p)\hat{a}_n - i g_n \beta_n \hat{a}_{-n}^\dagger - \frac{\kappa_n}{2} \hat{a}_n \\ & + \sqrt{\kappa_{n,e}} \hat{a}_{in}(t) e^{i\omega_p t} + \sqrt{\kappa_{n,i}} \hat{a}_{in,i}(t) e^{i\omega_p t}.\end{aligned} \tag{3}$$

Our cavity has equally spaced modes in the frequency domain (assuming negligible GVD over the frequency range of interest). To capture this mode structure in our model, we introduce

$\Omega_n = n\Omega$, where Ω is the free spectral range (FSR) of the cavity. Defining Δ_n as the amount of detuning of the nth mode from the resonance frequency, we have $\omega_n = \Omega_n + \Delta_n + \omega_p$. To understand the dynamics for each of the frequency modes, we think in terms of the slowly varying operators which we define as $\hat{a}_{n,s}(t) = e^{i\Omega_n t}\hat{a}_n(t)$ and $\hat{a}_{in,s}(t) = \hat{a}_{in}(t)e^{i\omega_p t}$. Then, for slowly varying operators, the equation of motion becomes

$$\begin{aligned}\frac{d\hat{a}_{n,s}}{dt} &= -i\Delta_n\hat{a}_{n,s} - ig_n\beta_n\hat{a}^\dagger_{-n,s} - \frac{\kappa_n}{2}\hat{a}_{n,s} \\ &\quad +\sqrt{\kappa_{n,e}}\hat{a}_{in,s}(t)e^{in\Omega t} + \sqrt{\kappa_{n,i}}\hat{a}_{in,i,s}(t)e^{in\Omega t} \\ &= -i\Delta_n\hat{a}_{n,s} - ig_n\beta_n\hat{a}^\dagger_{-n,s} - \frac{\kappa_n}{2}\hat{a}_{n,s} \\ &\quad +\sqrt{\kappa_{n,e}}\hat{a}_{in,s,n} + \sqrt{\kappa_{n,i}}\hat{a}_{in,i,s,n}. \end{aligned} \tag{4}$$

In our squeezing measurement, we are interested in the mode closest to the frequency of the local oscillator (ω_p). Since the bandwidth of our homodyne detector (450 MHz) is much smaller than the FSR of our cavity (~5.7 GHz), in the frame of local oscillator frequency the detector cannot capture the dynamics of other frequency modes because it is rapidly oscillating.

Now, we consider the equation of motion for the single mode closest to the frequency of ω_p. Dropping the subscript for the simplicity of notation we get,

$$\frac{d\hat{a}}{dt} = -i\Delta\hat{a} - 2ig\beta\hat{a}^\dagger - \frac{\kappa}{2}\hat{a} + \sqrt{\kappa_e}\hat{a}_{in} + \sqrt{\kappa_i}\hat{a}_{in,i}. \tag{5}$$

In the frequency domain, the equation of motion becomes

$$\begin{aligned}-i\omega\hat{a}(\omega) &= -i\Delta\hat{a}(\omega) - 2ig\beta\hat{a}^\dagger(-\omega) \\ &-\frac{\kappa}{2}\hat{a}(\omega) + \sqrt{\kappa_e}\hat{a}_{in}(\omega) + \sqrt{\kappa_i}\hat{a}_{in,i}(\omega)\end{aligned} \tag{6}$$

$$\begin{aligned}i\omega\hat{a}^\dagger(\omega) &= i\Delta\hat{a}^\dagger(\omega) + 2ig\beta^*\hat{a}(-\omega) \\ &-\frac{\kappa}{2}\hat{a}^\dagger(\omega) + \sqrt{\kappa_e}\hat{a}^\dagger_{in}(\omega) + \sqrt{\kappa_i}\hat{a}^\dagger_{in,i}(\omega)\end{aligned} \tag{7}$$

where Eq.(7) is the hermitian conjugate form of Eq.(6).

2.2 Squeezing at the output of the cavity

We follow the derivations from (*38, 39*) and calculate squeezing in the good cavity limit (i.e., $\kappa \sim \kappa_e$). We will include effect of intrinsic loss (κ_i) in the escape efficiency factor defined in the following section. Using the boundary condition $\hat{a}_{out}(\omega) = \sqrt{\kappa}\hat{a}(\omega) - \hat{a}_{in}(\omega)$ and the equations of motion (Eq. (6) and Eq. (7)), we could express $\hat{a}_{out}(\omega)$ in terms of $\hat{a}_{in}(\omega)$

$$\hat{a}_{out}(\omega) = \frac{\frac{\kappa^2}{4} + \omega^2 + 4g|\beta|^2}{(\frac{\kappa}{2} - i\omega)^2 - 4g^2|\beta|^2}\hat{a}_{in}(\omega) - \frac{2ig\beta\kappa}{(\frac{\kappa}{2} + i\omega)^2 - 4g^2|\beta|^2}\hat{a}^{\dagger}_{in}(-\omega) \quad (8)$$

$$\hat{a}^{\dagger}_{out}(\omega) = \frac{\frac{\kappa^2}{4} + \omega^2 + 4g|\beta|^2}{(\frac{\kappa}{2} + i\omega)^2 - 4g^2|\beta|^2}\hat{a}^{\dagger}_{in}(\omega) + \frac{2ig\beta^*\kappa}{(\frac{\kappa}{2} + i\omega)^2 - 4g^2|\beta|^2}\hat{a}_{in}(-\omega). \quad (9)$$

We define the two quadratures of the output as $\hat{X}_{out} = \hat{a}^{\dagger}_{out} + \hat{a}_{out}$ and $\hat{Y}_{out} = i(\hat{a}^{\dagger}_{out} - \hat{a}_{out})$. The variance of $\hat{X}_{out}$ is $\langle \hat{X}_{out}(\omega), \hat{X}_{out}(\omega')\rangle = \langle \hat{X}_{out}(\omega)\hat{X}_{out}(\omega')\rangle - \langle \hat{X}_{out}(\omega)\rangle\langle \hat{X}_{out}(\omega')\rangle$. Using $\langle \hat{X}_{out}(\omega)\rangle = 0$ and defining the phase of the output as ϕ_{out} (i.e., $\hat{a}_{out}(\omega) \rightarrow e^{i\phi_{out}}\hat{a}_{out}(\omega)$) we get

$$\begin{aligned}\langle \hat{X}_{out}(\omega), \hat{X}_{out}(\omega')\rangle = \; & \langle \hat{X}_{out}(\omega)\hat{X}_{out}(\omega')\rangle \\ = \; & e^{-2i\phi_{out}}\langle \hat{a}^{\dagger}_{out}(\omega)\hat{a}^{\dagger}_{out}(\omega')\rangle \\ & + e^{2i\phi_{out}}\langle \hat{a}_{out}(\omega)\hat{a}_{out}(\omega')\rangle \\ & + 2\langle \hat{a}^{\dagger}_{out}(\omega)\hat{a}_{out}(\omega')\rangle \\ & + \delta(\omega - \omega') \end{aligned} \quad (10)$$

where we used $[\hat{a}_{out}(\omega), \hat{a}^{\dagger}_{out}(\omega')] = \delta(\omega - \omega')$. We can calculate each of the terms in Eq.(10) and get

$$\langle \hat{a}_{out}(\omega)\hat{a}_{out}(\omega')\rangle = \frac{-2ig\beta\kappa[\frac{\kappa^2}{4} + \omega^2 + 4g|\beta|^2]}{[(\frac{\kappa}{2} - i\omega)^2 - 4g^2|\beta|^2][(\frac{\kappa}{2} - i\omega')^2 - 4g^2|\beta|^2]}\delta(\omega + \omega') \quad (11)$$

$$\langle \hat{a}^{\dagger}_{out}(\omega)\hat{a}^{\dagger}_{out}(\omega')\rangle = \frac{2ig\beta^*\kappa[\frac{\kappa^2}{4}+\omega'^2+4g|\beta|^2]}{[(\frac{\kappa}{2}+i\omega)^2-4g^2|\beta|^2][(\frac{\kappa}{2}+i\omega')^2-4g^2|\beta|^2]}\delta(\omega+\omega') \tag{12}$$

$$\langle \hat{a}^{\dagger}_{out}(\omega)\hat{a}_{out}(\omega')\rangle = \frac{4g^2|\beta|^2\kappa^2}{[(\frac{\kappa}{2}+i\omega)^2-4g^2|\beta|^2][(\frac{\kappa}{2}-i\omega')^2-4g^2|\beta|^2]}\delta(\omega-\omega'). \tag{13}$$

Plugging in Eq.(11-13) to Eq.(10) and integrating over ω', we arrive at

$$\begin{aligned} S_{XX}(\omega) = 1 &+ \frac{g|\beta|\kappa}{(\frac{\kappa}{2}-2g|\beta|)^2+\omega^2}(2+2\sin(2\phi_{out}+\phi_\beta)) \\ &+ \frac{g|\beta|\kappa}{(\frac{\kappa}{2}+2g|\beta|)^2+\omega^2}(-2+2\sin(2\phi_{out}+\phi_\beta)) \end{aligned} \tag{14}$$

where we defined $\beta = |\beta|e^{i\phi_\beta}$. We can see that we could get minimum and maximum at $2\phi_{out}+\phi_\beta = \pm\frac{\pi}{6}$. Since the threshold condition for oscillation implies $|\beta|_{thr} = \frac{\kappa}{4g}$, we can further simplify our form into

$$S_\pm(\omega) = 1 \pm \frac{4\frac{|\beta|}{|\beta|_{thr}}}{(1\mp\frac{|\beta|}{|\beta|_{thr}})^2+4(\frac{\omega}{\kappa})^2}. \tag{15}$$

Here, $+$ and $-$ indicates anti-squeezing and squeezing respectively.

2.3 Including the loss in our system and its effect on measured squeezing

Loss after the squeezing section diminishes the amount of squeezing and anti-squeezing that could be measured. For every component in our system with optical transmission less than unity ($\eta < 1$), we define an equivalent optical beamsplitter with the splitting ratio $\eta : (1-\eta)$ where the output is η times the input and $(1-\eta)$ times vacuum. The detected field quadrature after this beamsplitter could then be written as $\hat{X}_{out,det} = \sqrt{\eta}\hat{X}_{out} + \sqrt{1-\eta}\hat{X}_{vac}$. The source of vacuum noise is uncorrelated with the cavity output, and thus the field quadrature spectral

density is $S_{XX,det}(\omega) = \eta S_{XX,out}(\omega) + (1-\eta)S_{XX,vac}(\omega)$ where $S_{XX,vac}(\omega) = 1$ is the shot noise.

Our system has three major sources of loss: inefficient extraction efficiency of the cavity (low escape efficiency), transmission at the output of the chip to free space interface, and quantum efficiency of the photodetector. We define an equivalent beamsplitter for each of these components where the transmission coefficient are $\rho = \frac{\kappa_e}{\kappa}$ (escape efficiency), T (transmission from the output of the cavity to the detector), and ϵ (quantum efficiency of our detectors). Then, we may write $S_{\pm,meas}(\omega) = \eta_{tot} S_{\pm}(\omega) + (1-\eta_{tot})$ where $\eta_{tot} = \rho T \epsilon$ following from the product of beamsplitters. Finally, from Eq. (15) we get the form for the measured squeezing and antisqueezing

$$S_{\pm,meas} = \quad 1 \pm \rho T \epsilon \frac{4\sqrt{\frac{P}{P_{th}}}}{(1 \mp \sqrt{\frac{P}{P_{th}}})^2 + 4(\frac{\omega}{\kappa})^2}. \tag{16}$$

3 Laser noise theory and measurement

3.1 Theory of laser noise

The classical field amplitude of the output from a laser can be written in the general form,

$$\alpha(t) = \alpha e^{-i\omega_L t}(1 + N(t))e^{i\phi(t)} \tag{17}$$

where ω_L is the frequency of the laser, $N(t)$ and $\phi(t)$ are related to the intensity noise and phase noise respectively.

For a laser without excess intensity noise (i.e., $N(t) = 0$), we could relate the power spectral density of the phase noise term $\phi(t)$ to the linewidth of the laser. To see this, we calculate the autocorrelation of the classical field amplitude. Assuming that the random process $\phi(t)$ follows a gaussian probability distribution (*48*), we can write the autocorrelation as,

$$\langle \alpha(t)\alpha^*(0)\rangle \quad = |\alpha|t^2 \langle e^{i(\phi(t)-\phi(0)}\rangle$$

$$= |\alpha|^2 e^{-\langle(\phi(t)-\phi(0)\rangle^2/2} \tag{18}$$

where we used the property of the moments of gaussian probability distribution. We can further simplify the variance in terms of the power spectral density of the phase fluctuations,

$$\begin{aligned}
\langle(\phi(t)-\phi(0))^2\rangle \quad &= \langle((\phi(t)^2+\phi(0)^2-2\phi(t)\phi(0)\rangle \\
&= 2\langle\phi(0)^2\rangle - 2\langle\phi(t)\phi(0)\rangle \\
&= \frac{1}{\pi}\int_{-\infty}^{\infty} S_{\phi\phi}(\omega)(1-e^{-i\omega t})d\omega \\
&= \frac{2}{\pi}\int_{-\infty}^{\infty} S_{\phi\phi}(\omega)\sin^2(\omega t/2)d\omega. \qquad (19)
\end{aligned}$$

We may consider the case where the frequency noise is white. Defining the power spectral density of the frequency noise as $S_{\delta\delta}(\omega) = C$ where C is constant, the variance of the phase drift becomes

$$\langle(\phi(t)-\phi(0))^2\rangle = C|t|. \tag{20}$$

We note that the frequency noise and the phase noise have the relation $\omega^2 S_{\delta\delta}(\omega) = S_{\phi\phi}(\omega)$. The power spectrum is then

$$\begin{aligned}
S_{\alpha\alpha}(\omega) \quad &= \int_{-\infty}^{\infty} \langle\alpha(\tau)\alpha^*(0)\rangle e^{i\omega\tau}d\tau \\
&= \int_{-\infty}^{\infty} |\alpha|^2 e^{-\frac{C}{2}|t|} e^{i\omega\tau}d\tau \\
&= |\alpha|^2 \frac{4C}{C^2+4\omega^2} \qquad (21)
\end{aligned}$$

where the full-width at half-maximum (FWHM) is C, and the linewidth of the laser is $C/2\pi$ (Hz).

3.2 Intensity noise measurement

To check if there is no measurable excess intensity noise in addition to the shot noise from an ideal laser, we may perform a power sweep of the output of the laser and measure the power

spectral density (PSD) of the photodiode at different incident optical powers using the radio-frequency spectrum analyzer (RFSA) (Fig S7.(a)). The photocurrent operator of the photodiode when $\alpha(t)$ is incident with quantum fluctuation $\hat{a}_{vac}(t)$ is

$$\hat{I}(t) = G(\hat{a}_{vac}(t) + \alpha(t))^{\dagger}(\hat{a}_{vac}(t) + \alpha(t)) \tag{22}$$

where G is the gain of the detector (G is equal to e for an ideal photodetector with no amplification). Setting $G = 1$ for simplicity, the photocurrent power spectral density can then be written as

$$\begin{aligned} S_{II}(\Omega) &= \int_{-\infty}^{\infty} e^{i\omega\tau} \langle \hat{I}^{\dagger}(t)\hat{I}(t-\tau)\rangle d\tau \\ &= \eta^2|\alpha|^2 + \eta^2 \int_{-\infty}^{\infty} e^{i\omega\tau} |\alpha(t)|^2 |\alpha(t-\tau)|^2 d\tau \\ &= \eta^2|\alpha|^2 + \eta^2|\alpha|^4 S_{NN}(\Omega) \end{aligned} \tag{23}$$

where $S_{NN}(\Omega)$ is the PSD of the intensity noise fluctuations and η represents the non-unity efficiency of the detector. From Equation (23), we could see that the excess intensity noise has a quadratic dependence with respect to optical power incident on the photodetector. If there is measureable excess intensity noise on our laser, measured PSD of the photocurrent versus optical power incident on the detector would deviate from the ideal linear relation. In Fig S7.(d), we show that the power sweep shows a linear relation with coefficient of determination of $R^2 = 1$, verifying that our laser has no measureable excess intensity noise.

3.3 Phase noise measurement

To obtain an estimate of the phase noise of our laser, we send the output of our laser to a 2×2 fiber Mach-Zehnder interferometer (MZI) (Fig S7. (b)). The fiber MZI introduces a delay to the light passing through one of the arms due to the path length mismatch between the two arms. In the case where phase noise is small ($e^{i\phi(t)} \sim 1 + i\phi(t)$), we may write the output from one

of the two output ports as

$$\alpha_{out}(t) = \alpha \times (1 + i\phi(t) + e^{i\omega_L L/c}(1 + i\phi(t + L/c)) \tag{24}$$

where L is the path length difference and c is the speed of light. From equation (23), the power spectral density of the photocurrent of the detector can be written as

$$S_{II}(\Omega) = \eta^2|\alpha_{out}|^2 + \eta^2 \int_{-\infty}^{\infty} |\alpha_{out}(\Omega)|^2 |\alpha_{out}(\Omega')|^2 d\Omega' \tag{25}$$

where we used the $S_{AA}(\Omega) = \int_{-\infty}^{\infty} e^{i\omega\tau} A^*(t)A(t-\tau)d\tau = \int_{-\infty}^{\infty} A^*(\Omega)A(\Omega')d\Omega'$. To get $|\alpha_{out}(\Omega)|^2$, we first calculate $|\alpha_{out}(t)|^2$ and then take the fourier transform. Assuming $\phi^2 \ll \phi$, we arrive at the photocurrent power spectral density

$$S_{II}(\Omega) = \eta^2|\alpha_{out}|^2 + \eta^2|\alpha|^4 16 \sin^2(\omega_L L/c)\sin^2(\Omega L/2c) S_{\phi\phi}(\Omega) \tag{26}$$

where $S_{\phi\phi}(\Omega)$ is the PSD of the phase fluctuation. The shot noise level for this measurement is $\eta^2|\alpha_{out}|^2$ and it has a fixed relation set by the transfer function of the fiber MZI, $|\alpha_{out}|^2 = |\alpha|^2 \sin^2(\omega_L L/2c)$. Since we know the free spectral range (FSR) of our fiber MZI (67 MHz) from an independent measurement, by normalizing the PSD by the shot noise we can extract $S_{\phi\phi}(\Omega)$.

In a laboratory environment, fiber length may fluctuate and thus getting an accurate shot noise reference could be important for the phase noise measurement described in this section. We collect the DC voltage readout on the high-speed photodetector (Newport 12 GHz) when we take the PSD and use this as the reference for the shot noise. Figure S7.(e) compares the measured phase noise PSD to the theoretical prediction using the manufacturer-specified 100 Hz linewidth, showing good agreement.

4 Cavity transmission in the presence of intracavity parametric gain

We start from the equations of motion obtained from the hamiltonian of the system shown in Eq. (4). Assuming that we couple coherent light (α_{in}) into the cavity at the frequency around the resonance frequency of the mode $\hat{a}_n$, the coupled mode theory equation becomes

$$\frac{dA_n}{dt} = -i\Delta_n A_n - ig\beta A^*_{-n} - \frac{\kappa_n}{2}A_n + \sqrt{\kappa_{n,e}}\alpha_{in} \tag{27}$$

$$\frac{dA_{-n}}{dt} = -i\Delta_{-n} A_{-n} - ig\beta A^*_{n} - \frac{\kappa_n}{2}A_{-n} \tag{28}$$

where $A_{\pm n}$ is the fundamental field amplitude at $\omega_{\pm n}$. We look at the steady state and get the relation between the two field amplitudes

$$A^*_{-n} = \frac{-ig\beta^*}{i\Delta_n - \frac{\kappa_{-n}}{2}}A_n \tag{29}$$

where we assumed perfect spacing of the modes in the frequency domain and thus $\Delta_n = \Delta_{-n}$. Now, we assume $\kappa_n = \kappa_{-n}$ and use the boundary condition ($\alpha_{out} = \sqrt{\kappa_{n,e}}A_n - \alpha_{in}$) to get the output field (α_{out}) at around ω_n. Finally, we get the form for the transmission in the presence of intracavity parametric gain

$$\begin{aligned} T = & \; |\frac{\alpha_{out}}{\alpha_{in}}|^2 \\ = & \left|1 + \frac{\kappa_{n,e}(i\Delta - \frac{\kappa_n}{2})}{\Delta^2 + \frac{\kappa_n^2}{4} - g^2|\beta|^2}\right|^2. \end{aligned} \tag{30}$$

This formula is used to obtain the plot in Fig S5. (b). When $g|\beta| = 0$ (no gain), we see that the transmission is the well-known lorentzian form of the cold cavity transmission. The presence of parametric gain in the cavity has an effect of narrowing the linewidth of the mode. At a large enough gain, we may observe peak as opposed to dips due to amplification (see Fig S5.(b)). We also note that this measurement could be used to determine whether the cavity is

undercoupled or overcoupled. In the undercoupled situation, the dip of the transmission would go down to zero as we increase the gain (critically coupled) and become shallower with larger gain (overcoupled). In contrast, for the overcoupled case, the dip of the transmission would not go to zero but instead would only become shallower (more overcoupled).

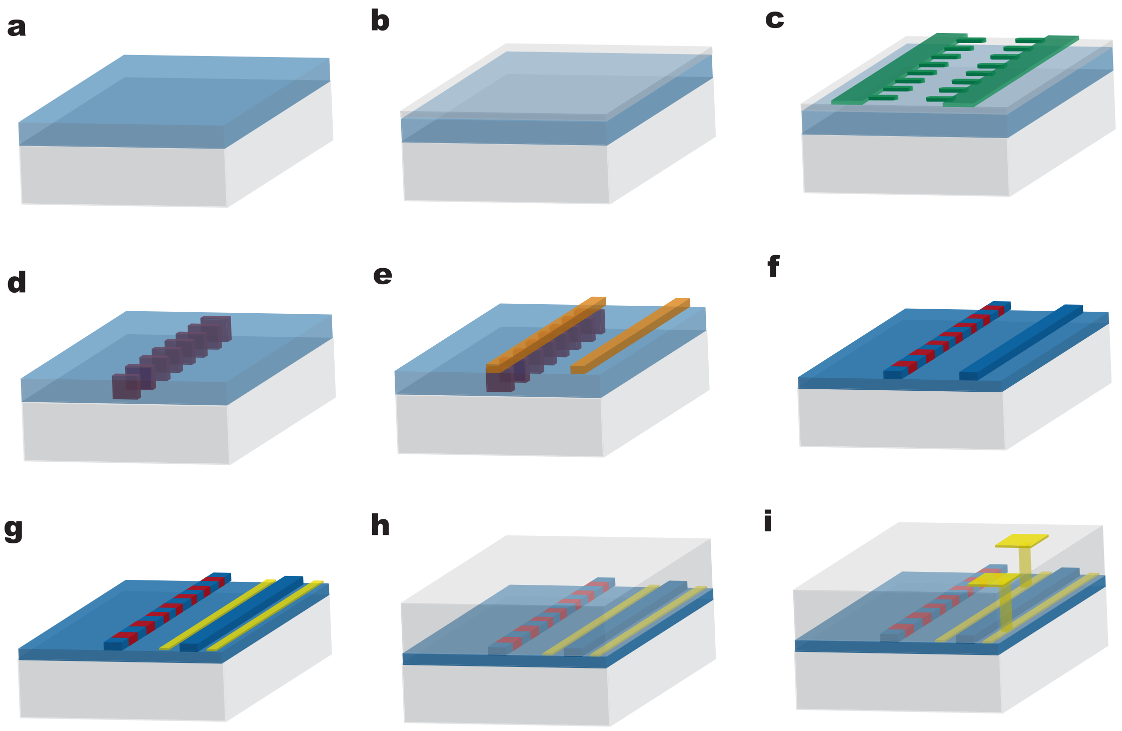

Fig S 1: **Fabrication procedure of the quantum photonic circuit.** **a**, We thin down a 700-nm thin-film lithium niobate on insulator chip to get 500-nm thickness. **b**, Before patterning electrodes for poling, we deposit 100 nm of SiO_2 using a high density plasma enhanced chemical vapor deposition (HDPCVD) tool. **c**, Using electron beam lithography and liftoff process, we fabricate chromium electrodes for poling. **d**, We apply high voltage pulses to periodically pole the LN and then remove the electrodes by chromium etchant. **e**, We pattern HSQ mask using electron beam lithography. **f**, Argon ion milling the LN and acid cleaning the chip, we get patterned LN waveguides. **g**, Then, we pattern 100-nm gold electrodes next to the waveguides, used to controlled the phase of the transmitted light, using electron beam lithography and liftoff. **h**, Using HDPCVD, we deposit 700 nm of SiO_2 for cladding. **i**, Finally, we make vias using inductively coupled plasma etch system and gold pads with electron beam lithography and liftoff process for the contact between the external probes and the 100-nm gold electrodes adjacent to waveguides.

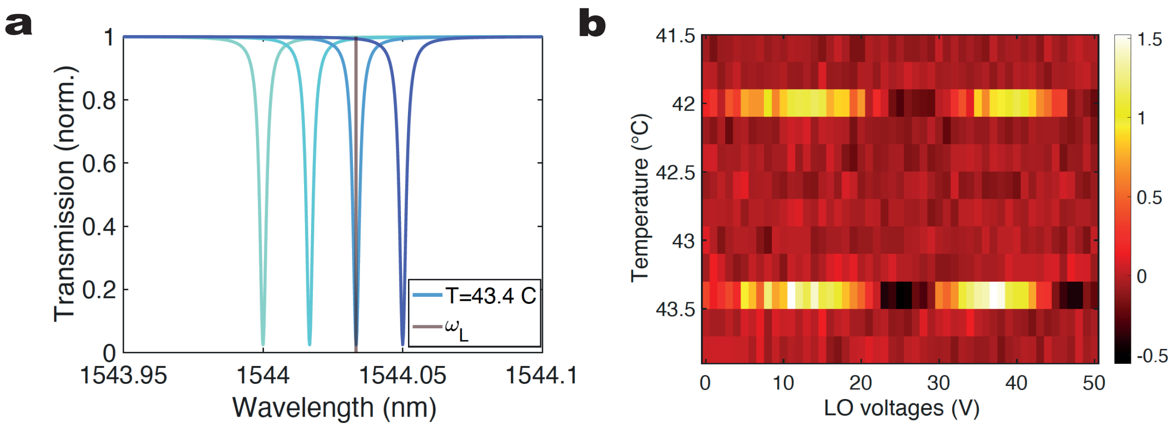

Fig S 2: **Cavity squeezing measurement with temperature stepping of the TEC setting and LO phase sweep. a**, Illustration of the mode resonance wavelength shift vs. temperature stepping of the TEC settings. Traces of different color (temperature stepping of approximately 0.4°C) indicate different temperature setting. At T = 43.4°C, the mode resonance wavelength aligns with the laser wavelength (ω_L). **b**, Photon noise level normalized to shot noise (dB scale) vs. DC voltage applied to the local oscillator (LO) phase shifter at different temperature settings of thermoelectric cooler (TEC).

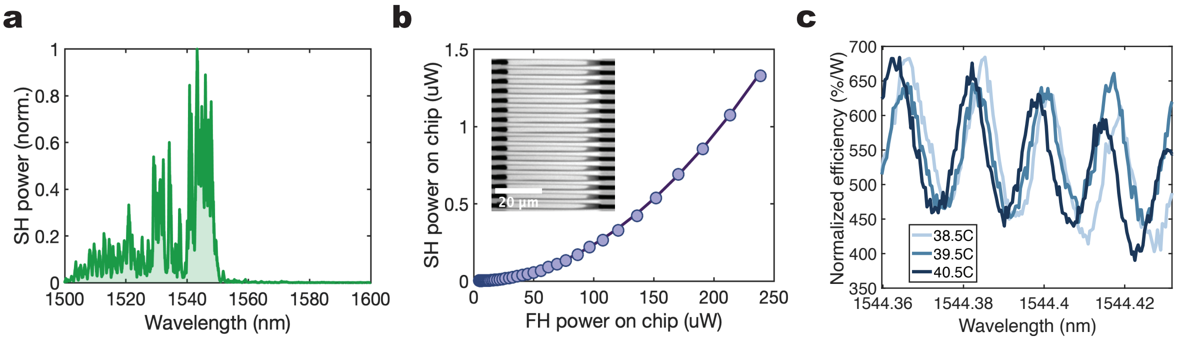

Fig S 3: **Waveguide second harmonic generator characterization.** **a**, SHG spectral response of the waveguide second harmonic generator. Deviation from the ideal sinc^2 response is presumably due to the inhomogeneity of waveguide geometry along the 1-cm-long section. **b**, On-chip SH power versus on-chip FH pump power of the diagnostic SHG waveguide at the peak SH response wavelength. A quadratic fit gives normalized conversion efficiency of 1000%/W. Inset is the second harmonic microscope image of the periodically poled section. We obtain a full-depth and uniform poling region over 30 μm in the lateral direction. **c**, Normalized efficiency at around the low-noise laser wavelength (1544.4 nm). Different colors indicate different temperature settings of the TEC. Oscillations with a period of approximately 20 pm originate from the Fabry-Perot cavity due to reflections at the interfaces between the waveguide and free space. The length of the cavity corresponds to the entire path length on the chip for this measurement.

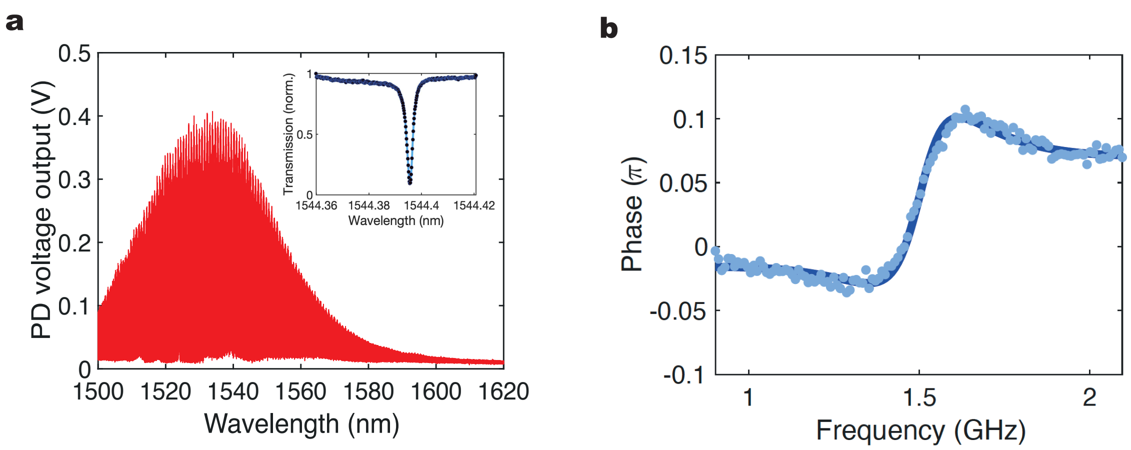

Fig S 4: **Cavity transmission profile and phase response of the mode.** **a**, Cavity transmission profile. The broad spectral response corresponds to the grating coupler response. Inset is the mode profile near the low-noise laser wavelength (1544.4 nm). A lorentzian fit on this mode gives Q_{tot} of 550k and Q_{int} of 950k assuming undercoupling. **b**, Phase response of a mode near 1544 nm. Light blue dots indicate data and the dark blue trace is the fit (see S.I. text).

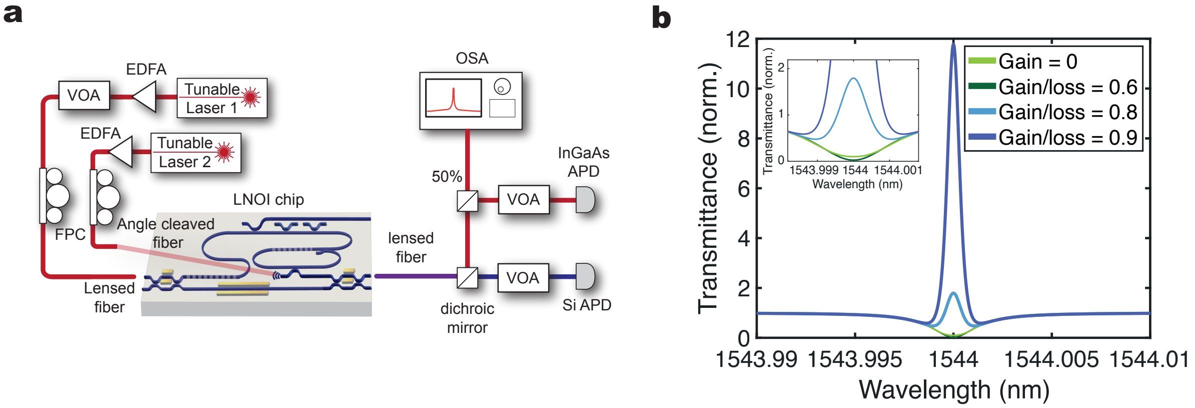

Fig S 5: **Optical parametric oscillator characterization setup and cavity transmission with gain theory.** **a**, Measurement setup used for characterizing the OPO. Tunable laser 1 is used for characterizing the threshold of our OPO. We use both lasers to measure the cavity transmission in the presence of parametric gain. **b**, Coupled mode theory plot of cavity transmittance at different gain levels. Different colors indicate different gain/loss ratio. As the gain increase the linewidth gets narrower and at a large enough gain we observe peak due to amplification. Here, we use measured quality factors of the mode near the low-noise laser wavelength.

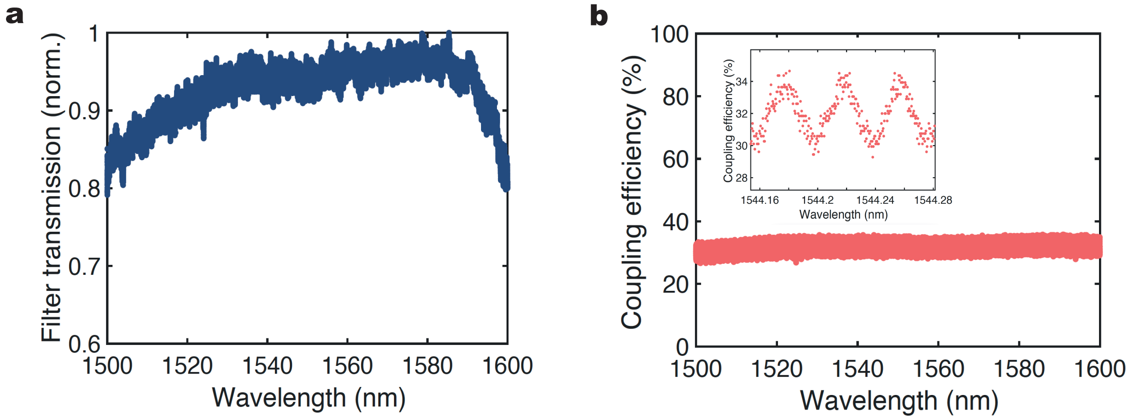

Fig S 6: **Fundamental harmonic transmission of the dichroic beamsplitter.** **a**, Transmission (normalized by the maximum transmission at around 1580 nm) of the dichroic beamsplitter at the fundamental harmonic verses wavelength. Assuming the maximum transmission data point has 100% transmission, the transmission at the low-noise laser wavelength (1544.4 nm) is roughly 94±2%. **b**, Lensed fiber to waveguide coupling efficiency versus wavelength. The coupling efficiency does not show substantial wavelength dependence from 1500 nm to 1600 nm.

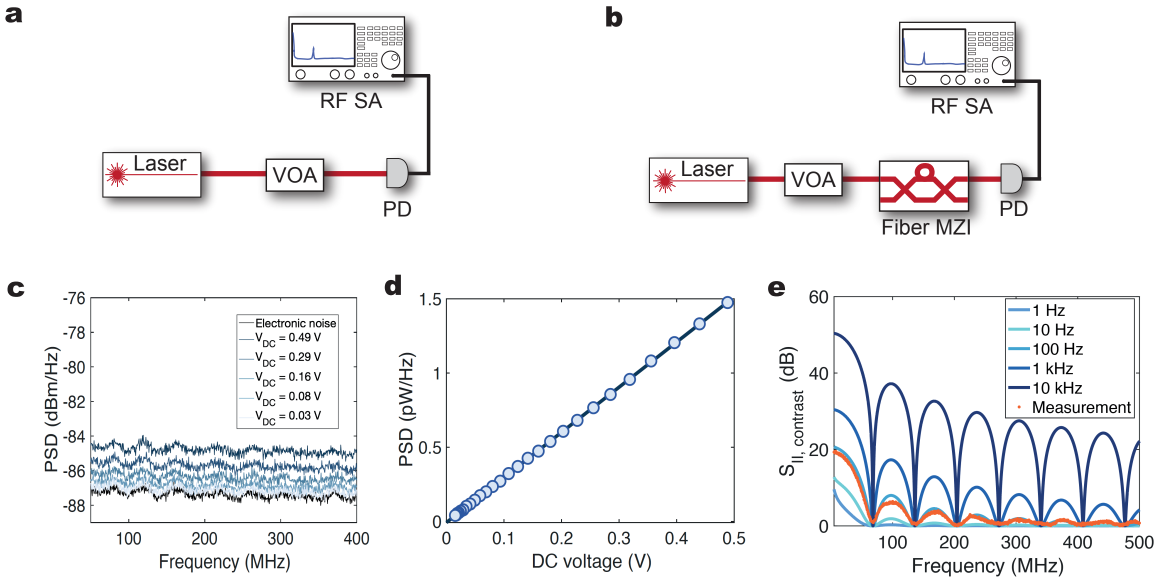

Fig S 7: **Laser noise characterization.** **a.** Intensity noise characterization setup. The output of the laser goes through a variable optical attenuator (VOA) to vary the optical power incident on the photodiode (PD). The photocurrent power spectral density (PSD) from the photodetector is measured using the radio frequency spectrum analyzer (RFSA) at different incident optical powers. **b.** Phase noise characterization setup. The output of the laser goes through a fiber MZI that introduces a delay (depicted as a circle on the top path) in one of the two paths. For our fiber MZI, the path length difference was 3 m, corresponding to the free spectral range (FSR) of 67 MHz. The photocurrent PSD is measured using the RFSA. **c**, Measured photocurrent power spectral density (PSD) spectrum at different optical power incident on the photodetector. **d**, Measured photocurrent power spectral density (PSD) averaged from 10 MHz to 500 MHz versus DC voltage read of the photodetector (electronic noise subtracted). The linear fit gives $R^2 = 1$, showing that there is no measurable excess intensity noise. **e**, Measured PSD spectrum after the output of the low-noise laser goes through a fiber MZI with a free spectral range of 67 MHz. Orange is the measurement data and blue traces are theory plots assuming different linewidth of the laser. The measured data aligns with the theory plot assuming the laser linewidth of 100 Hz, which is the documented value from manufacturer.

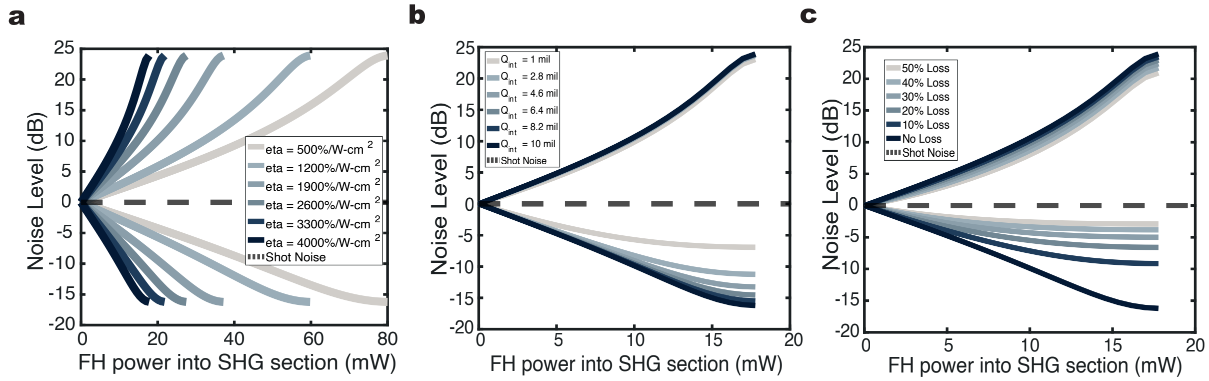

Fig S 8: **Anticipated squeezing and anti-squeezing with (a) different nonlinear conversion efficiencies, (b) intrinsic cavity quality factors, and (c) aggregate losses.** (a) assumes no aggregate loss after squeezed light generation with a fixed total quality factor at 200*k* and an intrinsic quality factor of 10 million. For (b), we set the total quality factor at 200*k* and the normalized nonlinear conversion efficiency at the ideal 4000%/W-cm^2. (c) assumes the ideal normalized nonlinear conversion efficiency of 4000%/W-cm^2 and a total quality factor of 200*k* with an intrinsic quality factor of 10 million.

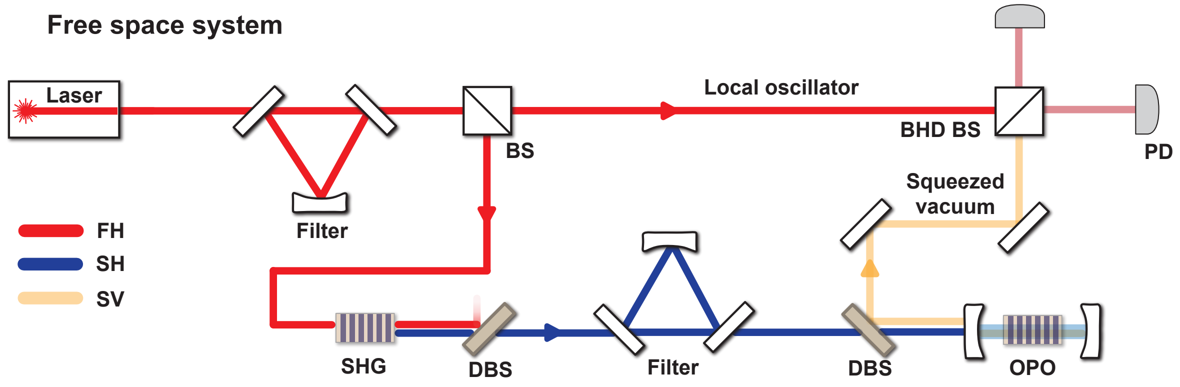

Fig S 9: **Free space system for generating and analyzing squeezed light using a sub-threshold optical parametric oscillator.** Free space system for generating and analyzing squeezed vacuum state of light from a sub-threshold optical parametric oscillator (OPO). Output of the laser at the fundamental harmonic (FH) goes through a filter to get a clean mode profile and gets split into two paths using a beamsplitter (BS). One path goes to the second harmonic generator (SHG) section to prepare pump light for the OPO while the light on the other path acts as a local oscillator (LO). The remaining FH light after SHG is filtered out using a dichroic beamsplitter (DBS). Second harmonic light goes through a filter for clean mode profile and pumps the OPO below oscillation threshold. The output from the OPO at the FH is a squeezed vacuum state of light. The generated squeezed light is sent to the 50:50 BS (BHD BS) and superimposed with the LO. The two outputs that are balanced in intensity are sent to the photodiodes. The fluctuations of the difference between the two photocurrents from the photodiodes are measured using a radio frequency spectrum analyzer (RFSA).

REFERENCES AND NOTES

1. E. Pelucchi, G. Fagas, I. Aharonovich, D. Englund, E. Figueroa, Q. Gong, H. Hannes, J. Liu, C.-Y. Lu, N. Matsuda, J.-W. Pan, F. Schreck, F. Sciarrino, C. Silberhorn, J. Wang, K. D. Jöns, The potential and global outlook of integrated photonics for quantum technologies. *Nat. Rev. Phys.* **4**, 194–208 (2022).

2. E. Polino, M. Valeri, N. Spagnolo, F. Sciarrino, Photonic quantum metrology. *AVS Quantum Sci.* **2**, 024703 (2020).

3. D. F. Walls, Squeezed states of light. *Nature* **306**, 141–146 (1983).

4. G. Moody, V. J. Sorger, D. J. Blumenthal, P. W. Juodawlkis, W. Loh, C. Sorace-Agaskar, A. E. Jones, K. C. Balram, J. C. F. Matthews, A. Laing, M. Davanco, L. Chang, J. E. Bowers, N. Quack, C. Galland, I. Aharonovich, M. A. Wolff, C. Schuck, N. Sinclair, M. Lonar, T. Komljenovic, D. Weld, S. Mookherjea, S. Buckley, M. Radulaski, S. Reitzenstein, B. Pingault, B. Machielse, D. Mukhopadhyay, A. Akimov, A. Zheltikov, G. S. Agarwal, K. Srinivasan, J. Lu, H. X. Tang, W. Jiang, T. P. McKenna, A. H. Safavi-Naeini, S. Steinhauer, A. W. Elshaari, V. Zwiller, P. S. Davids, N. Martinez, M. Gehl, J. Chiaverini, K. K. Mehta, J. Romero, N. B. Lingaraju, A. M. Weiner, D. Peace, R. Cernansky, M. Lobino, E. Diamanti, L. T. Vidarte, R. M. Camacho, 2022 Roadmap on integrated quantum photonics. *J. Phys. Photonics* **4**, 012501 (2022).

5. K. Bongs, S. Bennett, A. Lohmann, Quantum sensors will start a revolution – if we deploy them right. *Nature* **617**, 672–675 (2023).

6. B. Yurke, Use of cavities in squeezed-state generation. *Phys. Rev. A* **29**, 408–410 (1984).

7. R. Schnabel, Squeezed states of light and their applications in laser interferometers. *Phys. Rep.* **684**, 1–51 (2017).

8. L.-A. Wu, H. Kimble, J. Hall, H. Wu, Generation of squeezed states by parametric down conversion. *Phys. Rev. Lett.* **57**, 2520–2523 (1986).

9. H. Vahlbruch, M. Mehmet, K. Danzmann, R. Schnabel, Detection of 15 db squeezed states of light and their application for the absolute calibration of photoelectric quantum efficiency. *Phys. Rev. Lett.* **117**, 110801 (2016).

10. J. Aasi, J. Abadie, B. P. Abbott, R. Abbott, T. D. Abbott, M. R. Abernathy, C. Adams, T. Adams, P. Addesso, R. X. Adhikari, C. Affeldt, O. D. Aguiar, P. Ajith, B. Allen, E. A. Ceron, D. Amariutei, S. B. Anderson, W. G. Anderson, K. Arai, M. C. Araya, C. Arceneaux, S. Ast, S. M. Aston, D. Atkinson, P. Aufmuth, C. Aulbert, L. Austin, B. E. Aylott, S. Babak, P. T. Baker, S. Ballmer, Y. Bao, J. C. Barayoga, D. Barker, B. Barr, L. Barsotti, M. A. Barton, I. Bartos, R. Bassiri, J. Batch, J. Bauchrowitz, B. Behnke, A. S. Bell, C. Bell, G. Bergmann, J. M. Berliner, A. Bertolini, J. Betzwieser, N. Beveridge, P. T. Beyersdorf, T. Bhadbhade, I. A. Bilenko, G. Billingsley, J. Birch, S. Biscans, E. Black, J. K. Blackburn, L. Blackburn, D. Blair, B. Bland, O. Bock, T. P. Bodiya, C. Bogan, C. Bond, R. Bork, M. Born, S. Bose, J. Bowers, P. R. Brady, V. B. Braginsky, J. E. Brau, J. Breyer, D. O. Bridges, M. Brinkmann, M. Britzger, A. F. Brooks, D. A. Brown, D. D. Brown, K. Buckland, F. Brckner, B. C. Buchler, A. Buonanno, J. Burguet-Castell, R. L. Byer, L. Cadonati, J. B. Camp, P. Campsie, K. Cannon, J. Cao, C. D. Capano, L. Carbone, S. Caride, A. D. Castiglia, S. Caudill, M. Cavagli, C. Cepeda, T. Chalermsongsak, S. Chao, P. Charlton, X. Chen, Y. Chen, H.-S. Cho, J. H. Chow, N. Christensen, Q. Chu, S. S. Y. Chua, C. T. Y. Chung, G. Ciani, F. Clara, D. E. Clark, J. A. Clark, M. C. Junior, D. Cook, T. R. Corbitt, M. Cordier, N. Cornish, A. Corsi, C. A. Costa, M. W. Coughlin, S. Countryman, P. Couvares, D. M. Coward, M. Cowart, D. C. Coyne, K. Craig, J. D. E. Creighton, T. D. Creighton, A. Cumming, L. Cunningham, K. Dahl, M. Damjanic, S. L. Danilishin, K. Danzmann, B. Daudert, H. Daveloza, G. S. Davies, E. J. Daw, T. Dayanga, E. Deleeuw, T. Denker, T. Dent, V. Dergachev, R. DeRosa, R. DeSalvo, S. Dhurandhar, I. Di Palma, M. Daz, A. Dietz, F. Donovan, K. L. Dooley, S. Doravari, S. Drasco, R. W. P. Drever, J. C. Driggers, Z. Du, J.-C. Dumas, S. Dwyer, T. Eberle, M. Edwards, A. Effler, P. Ehrens, S. S. Eikenberry, R. Engel, R. Essick, T. Etzel, K. Evans, M. Evans, T. Evans, M. Factourovich, S. Fairhurst, Q. Fang, B. F. Farr, W. Farr, M. Favata, D. Fazi, H. Fehrmann, D. Feldbaum, L. S. Finn, R. P. Fisher, S. Foley, E. Forsi, N. Fotopoulos, M. Frede, M. A. Frei, Z. Frei, A. Freise, R. Frey, T. T. Fricke, D. Friedrich, P. Fritschel, V. V. Frolov, M.-K. Fujimoto, P. J. Fulda, M. Fyffe, J. Gair, J. Garcia, N. Gehrels, G. Gelencser, L.

Á. Gergely, S. Ghosh, J. A. Giaime, S. Giampanis, K. D. Giardina, S. Gil-Casanova, C. Gill, J. Gleason, E. Goetz, G. Gonzlez, N. Gordon, M. L. Gorodetsky, S. Gossan, S. Goler, C. Graef, P. B. Graff, A. Grant, S. Gras, C. Gray, R. J. S. Greenhalgh, A. M. Gretarsson, C. Griffo, H. Grote, K. Grover, S. Grunewald, C. Guido, E. K. Gustafson, R. Gustafson, D. Hammer, G. Hammond, J. Hanks, C. Hanna, J. Hanson, K. Haris, J. Harms, G. M. Harry, I. W. Harry, E. D. Harstad, M. T. Hartman, K. Haughian, K. Hayama, J. Heefner, M. C. Heintze, M. A. Hendry, I. S. Heng, A. W. Heptonstall, M. Heurs, M. Hewitson, S. Hild, D. Hoak, K. A. Hodge, K. Holt, M. Holtrop, T. Hong, S. Hooper, J. Hough, E. J. Howell, V. Huang, E. A. Huerta, B. Hughey, S. H. Huttner, M. Huynh, T. Huynh-Dinh, D. R. Ingram, R. Inta, T. Isogai, A. Ivanov, B. R. Iyer, K. Izumi, M. Jacobson, E. James, H. Jang, Y. J. Jang, E. Jesse, W. W. Johnson, D. Jones, D. I. Jones, R. Jones, L. Ju, P. Kalmus, V. Kalogera, S. Kandhasamy, G. Kang, J. B. Kanner, R. Kasturi, E. Katsavounidis, W. Katzman, H. Kaufer, K. Kawabe, S. Kawamura, F. Kawazoe, D. Keitel, D. B. Kelley, W. Kells, D. G. Keppel, A. Khalaidovski, F. Y. Khalili, E. A. Khazanov, B. K. Kim, C. Kim, K. Kim, N. Kim, Y.-M. Kim, P. J. King, D. L. Kinzel, J. S. Kissel, S. Klimenko, J. Kline, K. Kokeyama, V. Kondrashov, S. Koranda, W. Z. Korth, D. Kozak, C. Kozameh, A. Kremin, V. Kringel, B. Krishnan, C. Kucharczyk, G. Kuehn, P. Kumar, R. Kumar, B. J. Kuper, R. Kurdyumov, P. Kwee, P. K. Lam, M. Landry, B. Lantz, P. D. Lasky, C. Lawrie, A. Lazzarini, A. Le Roux, P. Leaci, C.-H. Lee, H. K. Lee, H. M. Lee, J. Lee, J. R. Leong, B. Levine, V. Lhuillier, A. C. Lin, V. Litvine, Y. Liu, Z. Liu, N. A. Lockerbie, D. Lodhia, K. Loew, J. Logue, A. L. Lombardi, M. Lormand, J. Lough, M. Lubinski, H. Lck, A. P. Lundgren, J. Macarthur, E. Macdonald, B. Machenschalk, M. MacInnis, D. M. Macleod, F. Magaa-Sandoval, M. Mageswaran, K. Mailand, G. Manca, I. Mandel, V. Mandic, S. Mrka, Z. Mrka, A. S. Markosyan, E. Maros, I. W. Martin, R. M. Martin, D. Martinov, J. N. Marx, K. Mason, F. Matichard, L. Matone, R. A. Matzner, N. Mavalvala, G. May, G. Mazzolo, K. McAuley, R. McCarthy, D. E. McClelland, S. C. McGuire, G. McIntyre, J. McIver, G. D. Meadors, M. Mehmet, T. Meier, A. Melatos, G. Mendell, R. A. Mercer, S. Meshkov, C. Messenger, M. S. Meyer, H. Miao, J. Miller, C. M. F. Mingarelli, S. Mitra, V. P. Mitrofanov, G. Mitselmakher, R. Mittleman, B. Moe, F. Mokler, S. R. P. Mohapatra, D. Moraru, G. Moreno, T. Mori, S. R. Morriss, K. Mossavi, C. M. Mow-Lowry, C. L. Mueller, G. Mueller, S. Mukherjee, A. Mullavey, J. Munch, D. Murphy, P. G. Murray, A. Mytidis, D. Nanda Kumar, T. Nash, R.

Nayak, V. Necula, G. Newton, T. Nguyen, E. Nishida, A. Nishizawa, A. Nitz, D. Nolting, M. E. Normandin, L. K. Nuttall, J. O'Dell, B. O'Reilly, R. O'Shaughnessy, E. Ochsner, E. Oelker, G. H. Ogin, J. J. Oh, S. H. Oh, F. Ohme, P. Oppermann, C. Osthelder, C. D. Ott, D. J. Ottaway, R. S. Ottens, J. Ou, H. Overmier, B. J. Owen, C. Padilla, A. Pai, Y. Pan, C. Pankow, M. A. Papa, H. Paris, W. Parkinson, M. Pedraza, S. Penn, C. Peralta, A. Perreca, M. Phelps, M. Pickenpack, V. Pierro, I. M. Pinto, M. Pitkin, H. J. Pletsch, J. Pld, F. Postiglione, C. Poux, V. Predoi, T. Prestegard, L. R. Price, M. Prijatelj, S. Privitera, L. G. Prokhorov, O. Puncken, V. Quetschke, E. Quintero, R. Quitzow-James, F. J. Raab, H. Radkins, P. Raffai, S. Raja, M. Rakhmanov, C. Ramet, V. Raymond, C. M. Reed, T. Reed, S. Reid, D. H. Reitze, R. Riesen, K. Riles, M. Roberts, N. A. Robertson, E. L. Robinson, S. Roddy, C. Rodriguez, L. Rodriguez, M. Rodruck, J. G. Rollins, J. H. Romie, C. Rver, S. Rowan, A. Rdiger, K. Ryan, F. Salemi, L. Sammut, V. Sandberg, J. Sanders, S. Sankar, V. Sannibale, L. Santamara, I. Santiago-Prieto, G. Santostasi, B. S. Sathyaprakash, P. R. Saulson, R. L. Savage, R. Schilling, R. Schnabel, R. M. S. Schofield, D. Schuette, B. Schulz, B. F. Schutz, P. Schwinberg, J. Scott, S. M. Scott, F. Seifert, D. Sellers, A. S. Sengupta, A. Sergeev, D. A. Shaddock, M. S. Shahriar, M. Shaltev, Z. Shao, B. Shapiro, P. Shawhan, D. H. Shoemaker, T. L. Sidery, X. Siemens, D. Sigg, D. Simakov, A. Singer, L. Singer, A. M. Sintes, G. R. Skelton, B. J. J. Slagmolen, J. Slutsky, J. R. Smith, M. R. Smith, R. J. E. Smith, N. D. Smith-Lefebvre, E. J. Son, B. Sorazu, T. Souradeep, M. Stefszky, E. Steinert, J. Steinlechner, S. Steinlechner, S. Steplewski, D. Stevens, A. Stochino, R. Stone, K. A. Strain, S. E. Strigin, A. S. Stroeer, A. L. Stuver, T. Z. Summerscales, S. Susmithan, P. J. Sutton, G. Szeifert, D. Talukder, D. B. Tanner, S. P. Tarabrin, R. Taylor, M. Thomas, P. Thomas, K. A. Thorne, K. S. Thorne, E. Thrane, V. Tiwari, K. V. Tokmakov, C. Tomlinson, C. V. Torres, C. I. Torrie, G. Traylor, M. Tse, D. Ugolini, C. S. Unnikrishnan, H. Vahlbruch, M. Vallisneri, M. V. van der Sluys, A. A. van Veggel, S. Vass, R. Vaulin, A. Vecchio, P. J. Veitch, J. Veitch, K. Venkateswara, S. Verma, R. Vincent-Finley, S. Vitale, T. Vo, C. Vorvick, W. D. Vousden, S. P. Vyatchanin, A. Wade, L. Wade, M. Wade, S. J. Waldman, L. Wallace, Y. Wan, M. Wang, J. Wang, X. Wang, A. Wanner, R. L. Ward, M. Was, M. Weinert, A. J. Weinstein, R. Weiss, T. Welborn, L. Wen, P. Wessels, M. West, T. Westphal, K. Wette, J. T. Whelan, S. E. Whitcomb, A. G. Wiseman, D. J. White, B. F. Whiting, K. Wiesner, C. Wilkinson, P. A. Willems, L. Williams, R. Williams, T. Williams, J. L. Willis, B. Willke, M. Wimmer, L. Winkelmann, W. Winkler, C.

C. Wipf, H. Wittel, G. Woan, R. Wooley, J. Worden, J. Yablon, I. Yakushin, H. Yamamoto, C. C. Yancey, H. Yang, D. Yeaton-Massey, S. Yoshida, H. Yum, M. Zanolin, F. Zhang, L. Zhang, C. Zhao, H. Zhu, X. J. Zhu, N. Zotov, M. E. Zucker, J. Zweizig, Enhanced sensitivity of the ligo gravitational wave detector by using squeezed states of light. *Nat. Photonics* **7**, 613–619 (2013).

11. V. Peano, H. G. L. Schwefel, C. Marquardt, F. Marquardt, Intracavity squeezing can enhance quantum-limited optomechanical position detection through deamplification. *Phys. Rev. Lett.* **115**, 243603 (2015).

12. J. S. Levy, A. Gondarenko, M. A. Foster, A. C. Turner-Foster, A. L. Gaeta, M. Lipson, CMOS-compatible multiple-wavelength oscillator for on-chip optical interconnects. *Nat. Photonics* **4**, 37–40 (2010).

13. J. Lu, A. Al Sayem, Z. Gong, J. B. Surya, C.-L. Zou, H. X. Tang, Ultralow-threshold thin-film lithium niobate optical parametric oscillator. *Optica* **8**, 539–544 (2021).

14. T. P. McKenna, H. S. Stokowski, V. Ansari, J. Mishra, M. Jankowski, C. J. Sarabalis, J. F. Herrmann, C. Langrock, M. M. Fejer, A. H. Safavi-Naeini, Ultra-low-power second-order nonlinear optics on a chip. *Nat. Commun.* **13**, 4532 (2022).

15. S. Paesani, Y. Ding, R. Santagati, L. Chakhmakhchyan, C. Vigliar, K. Rottwitt, L. K. Oxenløwe, J. Wang, M. G. Thompson, A. Laing, Generation and sampling of quantum states of light in a silicon chip. *Nat. Phys.* **15**, 925–929 (2019).

16. V. D. Vaidya, L. G. Helt, R. Shahrokshahi, D. H. Mahler, M. J. Collins, K. Tan, J. Lavoie, A. Repingon, M. Menotti, N. Quesada, R. C. Pooser, A. E. Lita, T. Gerrits, S. W. Nam, Z. Vernon, Broadband quadrature-squeezed vacuum and nonclassical photon number correlations from a nanophotonic device. *Sci. Adv.* **6**, eaba9186 (2020).

17. J. M. Arrazola, V. Bergholm, K. Brdler, T. R. Bromley, M. J. Collins, I. Dhand, A. Fumagalli, T. Gerrits, A. Goussev, L. G. Helt, J. Hundal, T. Isacsson, R. B. Israel, J. Izaac, S. Jahangiri, R. Janik, N. Killoran, S. P. Kumar, J. Lavoie, A. E. Lita, D. H. Mahler, M. Menotti,

B. Morrison, S. W. Nam, L. Neuhaus, H. Y. Qi, N. Quesada, A. Repingon, K. K. Sabapathy, M. Schuld, D. Su, J. Swinarton, A. Szva, K. Tan, P. Tan, V. D. Vaidya, Z. Vernon, Z. Zabaneh, Y. Zhang, Quantum circuits with many photons on a programmable nanophotonic chip. *Nature* **591**, 54–60 (2021).

18. Y. Zhao, Y. Okawachi, J. K. Jang, X. Ji, M. Lipson, A. L. Gaeta, Near-degenerate quadrature-squeezed vacuum generation on a silicon-nitride chip. *Phys. Rev. Lett.* **124**, 193601 (2020).

19. Y. Zhang, M. Menotti, K. Tan, V. D. Vaidya, D. H. Mahler, L. G. Helt, L. Zatti, M. Liscidini, B. Morrison, Z. Vernon, Squeezed light from a nanophotonic molecule. *Nat. Commun.* **12**, 2233 (2021).

20. A. H. Safavi-Naeini, S. Gröblacher, J. T. Hill, J. Chan, M. Aspelmeyer, O. Painter, Squeezed light from a silicon micromechanical resonator. *Nature* **500**, 185–189 (2013).

21. D. Zhu, L. Shao, M. Yu, R. Cheng, B. Desiatov, C. J. Xin, Y. Hu, J. Holzgrafe, S. Ghosh, A. Shams-Ansari, E. Puma, N. Sinclair, C. Reimer, M. Zhang, M. Lonar, Integrated photonics on thin-film lithium niobate. *Adv. Opt. Photonics* **13**, 242–352 (2021).

22. A. Boes, L. Chang, C. Langrock, M. Yu, M. Zhang, Q. Lin, M. Loncar, M. Fejer, J. Bowers, A. Mitchell, Lithium niobate photonics: Unlocking the electromagnetic spectrum. *Science* **379**, eabj4396 (2023).

23. C. Wang, C. Langrock, A. Marandi, M. Jankowski, M. Zhang, B. Desiatov, M. M. Fejer, M. Lončar, Ultrahigh-efficiency wavelength conversion in nanophotonic periodically poled lithium niobate waveguides. *Optica* **5**, 1438–1441 (2018).

24. J. Lu, M. Li, C.-L. Zou, A. Al Sayem, H. X. Tang, Toward 1% single-photon anharmonicity with periodically poled lithium niobate microring resonators. *Optica* **7**, 1654–1659 (2020).

25. J. Mishra, T. P. McKenna, E. Ng, H. S. Stokowski, M. Jankowski, C. Langrock, D. Heydari, H. Mabuchi, A. H. Safavi-Naeini, M. M. Fejer, Mid-infrared nonlinear optics in thin-film lithium niobate on sapphire. *Optica* **8**, 921–924 (2021).

26. T. Park, H. S. Stokowski, V. Ansari, T. P. McKenna, A. Y. Hwang, M. M. Fejer, A. H. Safavi-Naeini, High-efficiency second harmonic generation of blue light on thin-film lithium niobate. *Opt. Lett.* **47**, 2706–2709 (2022).

27. E. Hwang, N. Harper, R. Sekine, L. Ledezma, A. Marandi, S. Cushing, Tunable and efficient ultraviolet generation with periodically poled lithium niobate. *Opt. Lett.* **48**, 3917–3920 (2023).

28. M. Jankowski, C. Langrock, B. Desiatov, A. Marandi, C. Wang, M. Zhang, C. R. Phillips, M. Lončar, M. M. Fejer, Ultrabroadband nonlinear optics in nanophotonic periodically poled lithium niobate waveguides. *Optica* **7**, 40–46 (2020).

29. M. Jankowski, J. Mishra, M. M. Fejer, Dispersion-engineered $\chi(2)$ nanophotonics: A flexible tool for nonclassical light. *J. Phys. Photonics* **3**, 042005 (2021).

30. J. Mishra, M. Jankowski, A. Y. Hwang, H. S. Stokowski, T. P. McKenna, C. Langrock, E. Ng, D. Heydari, H. Mabuchi, A. H. Safavi-Naeini, M. M. Fejer, Ultra-broadband mid-infrared generation in dispersion-engineered thin-film lithium niobate. *Opt. Express* **30**, 32752–32760 (2022).

31. C. Wang, M. Zhang, X. Chen, M. Bertrand, A. Shams-Ansari, S. Chandrasekhar, P. Winzer, M. Loncar, Integrated lithium niobate electro-optic modulators operating at cmos-compatible voltages. *Nature* **562**, 101–104 (2018).

32. O. T. Celik, C. J. Sarabalis, F. M. Mayor, H. S. Stokowski, J. F. Herrmann, T. P. McKenna, N. R. A. Lee, W. Jiang, K. K. S. Multani, A. H. Safavi-Naeini, High-bandwidth cmos-voltage-level electro-optic modulation of 780 nm light in thin-film lithium niobate. *Opt. Express* **30**, 23177–23186 (2022).

33. R. Nehra, R. Sekine, L. Ledezma, Q. Guo, R. M. Gray, A. Roy, A. Marandi, Few-cycle vacuum squeezing in nanophotonics. *Science* **377**, 1333–1337 (2022).

34. H. S. Stokowski, T. P. McKenna, T. Park, A. Y. Hwang, D. J. Dean, O. T. Celik, V. Ansari, M. M. Fejer, A. H. Safavi-Naeini, Integrated quantum optical phase sensor in thin film lithium niobate. *Nat. Commun.* **14**, 3355 (2023).

35. M. Zhang, B. Buscaino, C. Wang, A. Shams-Ansari, C. Reimer, R. Zhu, J. M. Kahn, M. Loncar, Broadband electro-optic frequency comb generation in a lithium niobate mi-croring resonator. *Nature* **568**, 373–377 (2019).

36. H. S. Stokowski, D. J. Dean, A. Y. Hwang, T. Park, O. T. Celik, M. Jankowski, C. Langrock, V. Ansari, M. M. Fejer, A. H. Safavi-Naeini, Integrated frequency-modulated optical parametric oscillator. arXiv:2307.04200 (2023).

37. L. Ledezma, A. Roy, L. Costa, R. Sekine, R. Gray, Q. Guo, R. Nehra, R. M. Briggs, A. Marandi, Octave-spanning tunable infrared parametric oscillators in nanophotonics. *Sci. Adv.* **9**, eadf9711 (2023).

38. M. Collett, C. Gardiner, Squeezing of intracavity and traveling-wave light fields produced in parametric amplification. *Phys. Rev. A* **30**, 1386–1391 (1984).

39. C. W. Gardiner, M. J. Collett, Input and output in damped quantum systems: Quantum stochastic differential equations and the master equation. *Phys. Rev. A* **31**, 3761–3774 (1985).

40. A. E. Siegman, *Lasers* (University Science Books, 1986).

41. Y. Zhao, J. K. Jang, X. Ji, Y. Okawachi, M. Lipson, A. L. Gaeta, Large regenerative parametric amplification on chip at ultra-low pump powers. *Optica* **10**, 819–825 (2023).

42. L. S. Madsen, F. Laudenbach, M. F. Askarani, F. Rortais, T. Vincent, J. F. F. Bulmer, F. M. Miatto, L. Neuhaus, L. G. Helt, M. J. Collins, A. E. Lita, T. Gerrits, S. W. Nam, V. D. Vaidya, M. Menotti, I. Dhand, Z. Vernon, N. Quesada, J. Lavoie, Quantum computational advantage with a programmable photonic processor. *Nature* **606**, 75–81 (2022).

43. M. Chen, N. C. Menicucci and O. Pfister, Experimental realization of multipartite entanglement of 60 modes of a quantum optical frequency comb. *Phys. Rev. Lett.* **112**, 120505 (2014).

44. W. Asavanant, Y. Shiozawa, S. Yokoyama, B. Charoensombutamon, H. Emura, R. N. Alexander, S. Takeda, J.-I. Yoshikawa, N. C. Menicucci, H. Yonezawa, A. Furusawa, Generation of time-domain-multiplexed two-dimensional cluster state. *Science* **366**, 373–376 (2019).

45. J. Chan, "Laser cooling of an optomechanical crystal resonator to its quantum ground state of motion," thesis, California Institute of Technology (2012).

46. J. F. Herrmann, V. Ansari, J. Wang, J. D. Witmer, S. Fan, A. H. Safavi-Naeini, Mirror symmetric on-chip frequency circulation of light. *Nat. Photonics* **16**, 603–608 (2022).

47. M. Zhang, C. Wang, R. Cheng, A. Shams-Ansari, M. Lončar, Monolithic ultra-high-*Q* lithium niobate microring resonator. *Optica* **4**, 1536–1537 (2017).

48. M. Lax, Classical noise. V. Noise in self-sustained oscillators. *Phys. Rev.* **160**, 290–307 (1967).
